# An inhibitor of apoptosis (SfIAP) interacts with SQUAMOSA promoter‐binding protein (SBP) transcription factors that exhibit pro‐cell death characteristics

**DOI:** 10.1002/pld3.81

**Published:** 2018-08-10

**Authors:** Ryan Kessens, Nick Sorensen, Mehdi Kabbage

**Affiliations:** ^1^ Department of Plant Pathology University of Wisconsin‐Madison Madison Wisconsin

**Keywords:** cell death, fumonisin B1, inhibitor of apoptosis, necrotrophic, SQUAMOSA promoter‐binding protein‐like, SQUAMOSA promoter‐binding protein

## Abstract

Despite the importance of proper cell death regulation across broad evolutionary distances, an understanding of the molecular machinery underpinning this fundamental process in plants remains largely elusive. This is despite its critical importance to development, homeostasis, and proper responses to stress. The identification of endogenous plant regulators of cell death has been hindered by the fact that many core regulators of cell death in animals are absent in plant genomes. Remarkably, numerous studies have shown that the ectopic expression of animal prosurvival genes in plants can suppress cell death imposed by many stresses. In this study, we capitalize on the ectopic expression of one of these animal prosurvival genes, an inhibitor of apoptosis from *Spodoptera frugiperda* (*SfIAP*), to identify novel cell death regulators in plants. A yeast two‐hybrid assay was conducted using SfIAP as bait to screen a tomato cDNA library. This screen identified several transcription factors of the SQUAMOSA promoter‐binding protein (SBP) family as potential SfIAP binding partners. We confirmed this interaction *in vivo* for our top two interactors, SlySBP8b and SlySBP12a, using coimmunoprecipitation. Interestingly, overexpression of *SlySBP8b* and *SlySBP12a* induced cell death in *Nicotiana benthamiana* leaves. Overexpression of these two transcription factors also induced the accumulation of reactive oxygen species and enhanced the growth of the necrotrophic pathogen *Alternaria alternata*. Fluorescence microscopy confirmed the nuclear localization of both SlySBP8b and SlySBP12a*,* while SlySBP12a was also localized to the ER membrane. These results suggest a prodeath role for SlySBP8b and SlySBP12a and implicate ER membrane tethering as a means of regulating SlySBP12a activity.

## 1 INTRODUCTION

Proper cell death regulation is a fundamental aspect of development and stress response that is conserved throughout all kingdoms of life (Allocati, Masulli, Di Ilio, & De Laurenzi, 2015). This process of genetically regulated cellular suicide is referred to as programmed cell death (PCD). Programmed cell death has been studied extensively in animal systems, and the results of these research efforts have led to major treatment advances for many human diseases (Fuchs & Steller, 2011). In contrast, our understanding of the biochemical pathways underlying PCD in plants is severely lacking. This is largely due to the absence of obvious orthologs of core regulators of apoptosis, a well‐studied form of PCD in animals (Kabbage, Kessens, Bartholomay, & Williams, 2017). While this has undoubtedly slowed progress on plant PCD research, it has also presented a unique opportunity for the discovery of novel cell death regulators in plant systems.

Apoptosis is a specific type of PCD characterized by distinct morphological and biochemical features (Kroemer et al., 2009). Apoptotic cell death in animals is executed through the activation of cysteine‐dependent aspartate‐specific proteases termed caspases. Caspases exist as inactive proenzymes that can be activated by external or internal cellular cues. Once activated, caspases execute an orderly demise of the cell by targeting negative regulators of apoptosis, cytoskeletal components, and other caspases (Parrish, Freel, & Kornbluth, 2013). Due to the terminal nature of apoptosis, caspases must be kept under multiple layers of regulation. The inhibitor of apoptosis (IAP) family is an important group of proteins that negatively regulate caspase activity. The defining feature of all IAPs is the presence of one or more baculovirus IAP repeat (BIR) domains, which are used by IAP proteins for substrate binding (Verhagen, Coulson, & Vaux, 2001). Additionally, some IAPs contain a really interesting new gene (RING) domain that serves as a functional E3 ubiquitin ligase domain. Inhibitor of apoptosis proteins can inhibit caspase activity by preventing procaspases from becoming active or by suppressing active caspases. This can be accomplished by simply blocking the active site pocket of a caspase or by utilizing the RING domain to ubiquitinate a caspase and mark it for proteasome‐mediated degradation (Feltham, Khan, & Silke, 2012; Gyrd‐Hansen & Meier, 2010).

Despite the fact that obvious orthologs of IAPs and caspases are absent in plant genomes, the ectopic expression of animal and viral apoptotic regulators in tobacco (*Nicotiana* spp.) and tomato (*Solanum lycopersicum*) modulate plant cell death. This was first reported nearly two decades ago when the expression of *Bax,* a mammalian proapoptotic gene absent in plant genomes, induced localized tissue collapse and cell death in *Nicotiana benthamiana* (Lacomme & Santa, 1999). Shortly thereafter, Dickman et al. (2001) demonstrated that expression of a viral *IAP* (*OpIAP*), as well as anti‐apoptotic members of the Bcl‐2 family, conferred resistance to a suite of necrotrophic fungal pathogens in *Nicotiana tabacum*. Pathogens with a necrotrophic lifestyle require dead host tissue for nutrient acquisition, and studies on *Cochliobolus victoriae, Sclerotinia sclerotiorum,* and *Fusarium* spp. revealed that these necrotrophic fungal pathogens hijack host cell death machinery to kill cells (Asai et al., 2000; Glenn et al., 2008; Kabbage, Williams, & Dickman, 2013; Lorang et al., 2012; Williams, Kabbage, Kim, Britt, & Dickman, 2011).

More recently, we showed that overexpression of an *IAP* from *Spodoptera frugiperda* (fall armyworm; *SfIAP*) in tobacco and tomato prevented cell death associated with a wide range of abiotic and biotic stresses (Kabbage, Li, Chen, & Dickman, 2010; Li, Kabbage, & Dickman, 2010). Tobacco and tomato lines expressing *SfIAP* had increased heat and salt stress tolerance, two abiotic stresses that induce cell death. These transgenic lines were also resistant to the fungal necrotroph *Alternaria alternata* and the mycotoxin fumonisin B1 (FB1) (Li et al., 2010). Fumonisin B1 is produced by some species of *Fusarium* and is a potent inducer of apoptosis in animal cells and apoptotic‐like PCD in plant cells (Gilchrist, 1997).

It has been over 15 years since it was first reported that overexpression of animal anti‐apoptotic regulators in plants conferred enhanced resistance against a wide assortment of necrotrophic pathogens. During this time, numerous studies have confirmed the efficacy of animal apoptotic regulators in plants without identifying the means by which these regulators function. In this study, we used an unbiased approach to identify *in planta* binding partners of SfIAP in tomato to better understand how this insect IAP is able to inhibit cell death and confer stress tolerance in plants. Yeast two‐hybrid and coimmunoprecipitation (CoIP) assays show that SfIAP interacts with members of the SQUAMOSA promoter‐binding protein (abbreviated SBP in tomato or SQUAMOSA promoter‐binding protein‐like in some other species) transcription factor family. Overexpression of two tomato SBPs, *SlySBP8b* and *SlySBP12a*, induced cell death in tobacco leaves accompanied by enhanced production of reactive oxygen species (ROS). Overexpression of *SlySBP8b* and *SlySBP12a* also created an environment that was more conducive to the growth of the necrotrophic fungal pathogen *A. alternata*. In summary, our findings uncover SlySBP8b and SlySBP12a as novel SfIAP binding partners that exhibit prodeath attributes.

## 2 MATERIALS AND METHODS

### 2.1 Plant material and growth conditions

*Nicotiana benthamiana* plants were grown on a 16‐hr light cycle (~50 microeinsteins m^−2^ s^−1^) at 26°C and ~60% humidity. *Nicotiana glutinosa* (PI 555510) and tomato (*Solanum lycopersicum* cv. Bonny Best) plants were grown on a 16‐hr light cycle (~100 microeinsteins m^−2^ s^−2^) at 22°C and ~60% humidity. The soil composition for all plants consisted of SunGro^®^ propagation mix and Sunshine^®^ coarse vermiculite in a 3:1 ratio. Plants were watered with deionized water supplemented with Miracle‐Gro^®^ all‐purpose fertilizer (1 g/L) as needed.

### 2.2 Plasmid construction

The full‐length open reading frames of *SlySBP‐like* (Solyc07g062980), *SlySBP4* (Solyc07g053810), *SlySBP6a* (Solyc03g114850), *SlySBP6c* (Solyc12g038520), *SlySBP8b* (Solyc01g090730), and *SlySBP12a* (Solyc01g068100) were amplified by PCR from cDNA collected from tomato inflorescence tissue (Supporting information Table S1). AttB1 and attB2 adapters were added to forward and reverse primers, respectively, to generate attB‐flanked amplicons suitable for Gateway™ Recombination Cloning (Invitrogen).

Amplicons were recombined into the entry vector pDONR™/Zeo using BP clonase II (Invitrogen). *SlySBP8b(NLS*_*mt*_ *)* and *SlySBP12a(NLS*_*mt*_ *)* constructs were generated using the Q5^®^ Site‐Directed Mutagenesis Kit (New England Biolabs). *SlySBP12a(ΔTMD)* and *TMD*_*SlySBP12a*_ were amplified from *SlySBP12a* in pDONR™/Zeo using the primers indicated in Supporting information Table S1 and recombined into pDONR™/Zeo. For overexpression in *N. benthamiana* leaves and tomato protoplasts, entry vectors were mixed with the desired pEarleyGate destination vectors (Earley et al., 2006) and recombined using LR clonase II (Invitrogen). pEarleyGate vectors drive transgene expression using a cauliflower mosaic virus 35S (35S) promoter and were used to generate N‐terminal yellow fluorescent protein (YFP; pEarleyGate104) or N‐terminal influenza hemagglutinin (HA; pEarleyGate201) fusions. All constructs were verified using Sangar sequencing before being transformed into *Agrobacterium tumefaciens* GV3101.

Plasmids for the yeast two‐hybrid screen were prepared as follows. *SfIAP*,* SfIAP*_*BIR1*_, and *luciferase* cDNAs were cloned into the bait vector pGilda under control of the *GAL1* promoter and in‐frame with an N‐terminal fusion of the *E. coli* LexA DNA binding protein (Takara Bio USA, Inc.). *Luciferase* (firefly luciferase from *Photinus pyralis*) was cut from an existing plasmid using a 5′‐Nco1 restriction site in the START codon and a 3′‐Not1 restriction site outside of the ORF and ligated into pGilda. Primers for *SfIAP* (GenBank: AF186378.1) and *SfIAP*_*BIR1*_ amplification were designed to place an EcoR1 site at the 5′ end and a BamH1 site at the 3′ end of the ORF. Primers used for amplification can be found in Supporting information Table S1. Amplicons were cut using these restriction enzymes and ligated into pGilda. Tomato cDNAs were expressed from the *GAL1* promoter with an N‐terminal fusion of the B42 activation protein in the pB42AD plasmid (Takara Bio USA, Inc.). Bait and prey library were sequentially transformed into EGY48 yeast using standard protocols.

### 2.3 Yeast two‐hybrid screening

Yeast containing bait and plasmid were plated on SD galactose (‐His/‐Trp/‐Leu) to induce gene expression and select for bait–prey interactions. After incubating at 28°C for ~5 days, colonies were pooled in 10 ml of sorbitol/phosphate buffer (1.2 M sorbitol, 0.1 M NaPO_4_, pH 7.5) per plate, pelleted, and resuspended in 2 ml of sorbitol/phosphate buffer supplemented with 500 U of lyticase (Sigma: L2524‐25KU) and 250 μg of RNase A. Yeast cells were incubated in the lyticase buffer for 3 hr at 37°C prior to plasmid recovery. Plasmid DNA was extracted using a Wizard Plus SV Miniprep kit (Promega) and a modified protocol. Briefly, 2.5 ml of lysis solution and 80 μl of alkaline protease solution were added to yeast protoplasts and incubated at room temperature for 10 min. Next, 3.5 ml of neutralization solution was added and cellular debris was pelleted by centrifugation. Supernatant was run through the provided columns and plasmid DNA eluted according to the manufacturer's instructions. Low‐cycle PCR was performed to amplify cDNA's from the prey library. Briefly, MyFi™ proofreading DNA polymerase (Bioline) and pB42AD forward and reverse primers (flanking the cDNA insertion site of pB42AD) were used to amplify cDNA's (Supporting information Table S1). A QIAquick PCR purification kit (Qiagen) was used to clean PCR products before sequencing.

### 2.4 Illumina sequencing and data analysis

Sequencing was performed by the Biotechnology Center at UW‐Madison using Illumina next‐generation sequencing with 100‐bp paired‐end reads. The sequencing data were uploaded to the Galaxy web platform, and we used the public server at *usegalaxy.org* to analyze the data (Afgan et al., 2016). Reads were groomed and trimmed to remove low‐quality bases and adapter sequences before alignment (Bolger, Lohse, & Usadel, 2014). Bait (pGilda) and prey (pB42AD) plasmid sequences were concatenated with the *Saccharomyces cerevisiae* reference genome (S288C_reference_sequence_R64‐2‐1_20150113) to create a FASTA file containing sources of plasmid and gDNA contamination. Reads were aligned to this file using Bowtie 2 (Langmead & Salzberg, 2012). Aligned reads (plasmid and gDNA) were discarded while unaligned reads were aligned to the tomato reference genome (Solgenomics: ITAG2.4) with Bowtie 2. Cufflinks (v2.2.1) was used to assemble transcripts from these aligned reads and calculate FPKM values for each locus (Trapnell et al., 2012). Enrichment scores for each locus were calculated using R Studio and scripts written in‐house (RStudio Team, 2016). Details of Galaxy pipeline, in‐house scripts, and complete dataset are available upon request.

### 2.5 Transient expression in *N. benthamiana* and *N. glutinosa*

Agrobacterium strain GV3101 was grown overnight in liquid LB supplemented with gentamycin and kanamycin (50 μg/ml) at 28°C with shaking. Cells were harvested by centrifugation, washed once with sterile deionized water, and resuspended in infiltration medium (10 mM MgSO_4_, 9 mM MES, 10 mM MgCl_2_, 300 μM acetosyringone, pH 5.7) to a final concentration of OD_600_ = 0.9. Cultures were incubated at room temperature for 4 hr before infiltration. *Nicotiana benthamiana* plants were infiltrated with a 1‐ml needleless syringe at 4–5 weeks of age with the two youngest and easily infiltratable leaves being used. *Nicotiana glutinosa* plants were infiltrated at 5–6 weeks of age with a single leaf being used on each plant, typically corresponding to the 4th or 5th true leaf. Plants were transformed at different ages due to differences in rate of growth between the two species.

For total protein extraction, leaf tissue was frozen in liquid nitrogen and ground in 3× Laemmli buffer (10% β‐mercaptoethanol). Samples were boiled for 10 min followed by centrifugation at 10,000 *g* for 5 min. Supernatants were removed and transferred to new tubes. Total proteins were separated by electrophoresis on a 12% Tris‐Glycine‐SDS polyacrylamide gel (Bio‐Rad). Proteins were transferred to a nitrocellulose membrane. Total protein was detected using Ponceau S stain. Epitope‐tagged proteins were detected by probing with α‐GFP (Cell Signaling 2955S) or α‐HA (Cell Signaling 3724S) primary antibodies. The α‐GFP antibody was detected using goat α‐mouse IgG conjugated to horseradish peroxidase (HRP) (Cell Signaling 7076P2) while the α‐HA antibody was detected using goat α‐rabbit IgG conjugated to HRP (Cell Signaling 7074P2). Amersham™ ECL™ reagent (GE Life Sciences) was used to detect HRP‐conjugated antibodies.

### 2.6 Transient transfection of tomato protoplasts

Mesophyll protoplasts form tomato cotyledons were isolated from 10‐day‐old plants using the Tape Sandwich method (Wu et al., 2009). A total of 6 μg of plasmid was used for each transfection with an equal ratio used for cotransfections. Transfections were performed using polyethylene glycol (PEG) as described previously (Yoo, Cho, & Sheen, 2007). Protoplasts were used for imaging the day after transfection.

### 2.7 Coimmunoprecipitation assays

Agrobacterium strains harboring free *35S:YFP* or *35S:YFP‐SfIAP* ^*M4*^ *(I332A)* were coinfiltrated with strains harboring *35S:HA‐SlySBP8b* or *35S:HA‐SlySBP12a*. A 7:2 ratio of YFP strains to HA strains was used due to relatively low accumulation of YFP‐SfIAP^M4^(I332A) protein compared to HA‐SlySBP8b and HA‐SlySBP12a. Approximately 40 hr post‐agroinfiltration, transformed leaves were collected and ground in liquid nitrogen to a fine powder. Extraction buffer (50 mM Tris‐HCl, 150 mM NaCl, 5 mM EDTA, 10% glycerol, 0.2% IGEPAL, and 1% plant protease inhibitor cocktail [Sigma]) was added at a concentration of 2 ml/g of leaf tissue. YFP‐tagged proteins were immunoprecipitated by incubating the lysate with α‐GFP magnetic agarose beads (GFP‐Trap_MA; Chromotek) for 2 hr at 4°C. Beads were washed three times in extraction buffer (w/o IGEPAL) and boiled in 30 μl of 2× SDS loading buffer before loading on duplicate 12% Tris‐Glycine‐SDS polyacrylamide gels (Bio‐Rad). Proteins were transferred to duplicate nitrocellulose membranes and probed with α‐GFP (Cell Signaling 2955S) or α‐HA (Cell Signaling 3724S) primary antibodies. The α‐GFP antibody was detected using goat α‐mouse IgG conjugated to HRP (Cell Signaling 7076P2) while the α‐HA antibody was detected using goat α‐rabbit IgG conjugated to HRP (Cell Signaling 7074P2). Amersham™ ECL™ reagent (GE Life Sciences) was used to detect HRP‐conjugated antibodies.

### 2.8 Confocal laser scanning microscopy

Confocal laser scanning microscopy was performed on a Zeiss ELYRA LSM780 inverted confocal microscope using a 40×, 1.1‐numerical aperture, water objective. Protoplasts were stained with DHE at a concentration of 5 μM by adding DHE directly to protoplast solution and imaging approximately 10 min after DHE addition. YFP fusions, chlorophyll autofluorescence, and DHE were excited with a 488 nm argon laser. YFP emission was detected between 502 and 542 nm, chlorophyll emission was detected between 657 and 724 nm, and DHE was detected between 606 and 659 nm. mCherry was excited with a 561 nm He–Ne laser, and emission was detected between 606 and 651 nm. Colocalization analysis was performed using the Coloc 2 package in ImageJ. A region of interest was selected on the image, and the analysis was performed using a PSF of 3.0 with 100 Costes randomizations.

### 2.9 Electrolyte leakage analysis

Cell death progression in *N. benthamiana* leaves was assessed by measuring ion leakage. Approximately 24 hr post‐agroinfiltration, eight leaf disks were collected from two leaves on the same plant and pooled into a single well of a 12‐well plate, representing a single biological replicate. Leaf disks were washed for 30 min in 4 ml of deionized water by rotating plates at 50 rpm at room temperature. Wash water was removed and replaced with 4 ml of fresh deionized water. Immediately after adding freshwater, the conductivity of the solution was recorded, representing the 24 hr post‐agroinfiltration measurement. The conductivity of the water was measured using an ECTestr 11^+^ MultiRange conductivity meter (Oakton) at the indicated time points.

### 2.10 DAB staining of *N. benthamiana* leaves

Staining solution was prepared by dissolving 3,3′‐diaminobenzidine (DAB; Sigma) in HCl at pH 2. Once dissolved, this solution was added to Na_2_HPO_4_ buffer (10 mM) for a final DAB concentration of 1 mg/ml. Tween 20 (0.05% v/v) was added, and the final pH was adjusted to 7.2. Whole leaves were collected, placed in petri dishes, submerged in DAB staining solution, and vacuum infiltrated. Plates were covered in aluminum foil and incubated at room temperature with shaking. After 4 hr, DAB staining solution was removed and replaced with clearing solution A (25% acetic acid, 75% ethanol). Leaves were heated at 80°C for 10 min to remove chlorophyll. Clearing solution A was removed and replaced with clearing solution B (15% acetic acid, 15% glycerol, 70% ethanol). Leaves were incubated in clearing solution B overnight at room temperature to remove residual chlorophyll.

### 2.11 *A. alternata* inoculation of *N. glutinosa* leaves

*Alternaria alternata* isolated from potato was provided by Dr. Amanda Gevens (University of Wisconsin, Madison, WI). For FB1 treatments, leaves were coinfiltrated with an Agrobacterium suspension containing the *35S:YFP* construct and 5 μM FB1 (Cayman Chemicals). Leaves were harvested from *N. glutinosa* plants one day after agroinfiltration. Detached leaves were placed adaxial‐side up in petri dishes (100 mm × 20 mm) containing three layers of wet filter paper. Five‐mm‐diameter agar plugs were collected from the edge of an actively growing fungal colony on potato dextrose agar. Leaves were wounded with a 1‐ml pipette tip along the midrib, and agar plugs were placed fungal‐side‐down on top of the wound. Inoculated leaves were kept at room temperature (~23°C) for the duration of the experiment. Lesion areas were recorded 3 days after fungal inoculation.

### 2.12 Image acquisition and analysis

All leaf images were taken using a Nikon D5500 camera with a Nikon AF‐S NIKKOR 18‐55 mm lens. Quantification of DAB staining intensity and fungal growth was performed using the Fiji package for ImageJ (Schindelin et al., 2012). For quantification of DAB staining intensity, the Colour Deconvolution package was used to isolate the DAB color channel for each DAB‐stained leaf (Ruifrok & Johnston, 2001). Staining intensity caused by *35S:YFP* expression on the left half of each leaf was subtracted from the staining intensity caused by *35S:HA‐SlySBP8b* or *35S:HA‐SlySBP12a* expression on the right half of the same leaf. Fungal lesions were quantified by tracing the periphery of the lesion and calculating the area within the periphery using ImageJ. Statistical analyses were performed using a one‐way analysis of variance (ANOVA) with Tukey's honest significant difference (HSD) test in R Studio (RStudio Team, 2016).

### 2.13 Identification of SBP cis‐elements in Arabidopsis genes

The online bioinformatic tool FindM was used to search the eukaryotic promoter database (EPD) for promoters containing the 5′‐CCGTAC(A/G)‐3′ cis‐element bound by the SBP domain of SBP transcription factors. Promoter regions were defined as the genomic region 1,000‐bp upstream of the transcription start site, and a bidirectional search mode was used. Genomic sequences used were from the TAIR 10 version of the Arabidopsis genome.

## 3 RESULTS

### 3.1 Identification of SfIAP binding partners in tomato

To identify putative binding partners of SfIAP from tomato, we performed a yeast two‐hybrid assay coupled with next‐generation sequencing using a method developed by Lewis et al. (2012) termed quantitative interactor screen sequencing (QIS‐Seq). This method enables the entire pool of interactors to be sequenced by pooling all yeast colonies together instead of individually sequencing each colony using Sanger sequencing. The high‐throughput nature of QIS‐Seq proved useful for screening multiple baits, including a negative control, against the library as well as sequencing the entire cDNA library itself (Supporting information Figure S1A).

SfIAP contains two BIR domains and a C‐terminal RING domain. The BIR1 domain and the RING domain are essential for complete SfIAP function in plants while the BIR2 domain is dispensable (Kabbage et al., 2010). Full‐length SfIAP and the BIR1 domain alone (SfIAP_BIR1_) were used as bait to screen a tomato cDNA library produced under stressed conditions. The SfIAP_BIR1_ construct was used to prolong transient interactions that can occur upon ubiquitination of substrates by the RING domain of full‐length SfIAP. Luciferase served as a negative control to account for non‐specific protein interactions and potential autoactivation of the selectable marker. The cDNA library itself was also sequenced to account for biases in transcript abundance.

Enrichment scores were calculated for each locus using the equation in Supporting information Figure S1B. A total of 13 putative interactors with enrichment scores of 50 or higher were identified in our screen (Table 1). Interestingly, this list contained six members of the SQUAMOSA promoter‐binding protein (SBP) family of transcription factors. Based on enrichment scores, the top interactor with full‐length SfIAP was SlySBP8b (95.7) while the top interactor with SfIAP_BIR1_ was SlySBP12a (98.7). Also present at lower enrichments were SlySBP4, ‐6a, ‐6c, and an unannotated homolog referred to as “SlySBP‐like” (Table 1).

**Table 1:** Enriched genes identified from QIS‐Seq using full‐length SfIAP or the BIR1 domain alone as bait

Locus ID	Annotation	FPKM values			Enrichment
		SfIAP	Luciferase	Library	
Solyc01g090730	SlySBP8b	66124.2	1434.5	13.2	95.7
Solyc02g071010	Chlorophyll a/b binding	52964.4	765.5	4551.3	89.6
Solyc05g005560	BURP‐domain containing	94.9	2.5	9.3	86.6
Solyc03g114850	SlySBP6a	626.3	44.6	1.1	86.6
Solyc07g062980	SlySBP‐like	1489.9	294.3	6.9	66.8
Solyc12g038520	SlySBP6c	43.2	8.9	2.3	63.2
Solyc07g053810	SlySBP4	576.7	171.2	11.0	53.4

Locus ID	Annotation	FPKM values			Enrichment
		SfIAP_BIR1_	Luciferase	Library	
Solyc01g068100	SlySBP12a	7378.1	42.8	12.1	98.7
Solyc06g073090	Ribosomal sub. interface	5669.4	196.7	61.1	92.3
Solyc01g080020	Xylanase inhibitor	85.7	4.4	7.0	83.7
Solyc01g090690	Elongation factor G	28.4	1.3	3.2	82.4
Solyc01g090730	SlySBP8b	14122.1	1434.5	13.2	81.5
Solyc01g094200	NAD‐dep. malic enzyme	54.5	2.0	9.4	79.7
Solyc12g038520	SlySBP6c	69.2	8.9	2.3	75.0
Solyc07g053810	SlySBP4	1031.9	171.2	11.0	70.9
Solyc07g062980	SlySBP‐like	1257.7	294.3	6.9	61.8
Solyc01g009750	Unknown Protein	69.1	10.2	33.2	52.4

FPKM: fragments per kilobase per million reads.

### 3.2 Induction of tissue death by SlySBP8b and SlySBP12a

SfIAP is known to inhibit apoptosis in *S. frugiperda* and suppress cell death when ectopically expressed in tomato and tobacco (Li et al., 2010; Muro, Hay, and Clem, 2002). Thus, we anticipated that SfIAP‐interacting partners in plants may be positive regulators of cell death. To narrow our list of candidate genes, we transiently overexpressed full‐length cDNA clones of the six tomato SBPs identified from our yeast two‐hybrid screen in *N. benthamiana* leaves and monitored these leaves for signs of tissue death. The generated cassettes contained an N‐terminal hemagglutinin (HA) tag and were driven by a cauliflower mosaic virus 35S promoter (35S). The structures of these SBPs are quite diverse; the SBP domain being the only domain conserved among all six members (Supporting information Figure S2).

**Figure 1:**
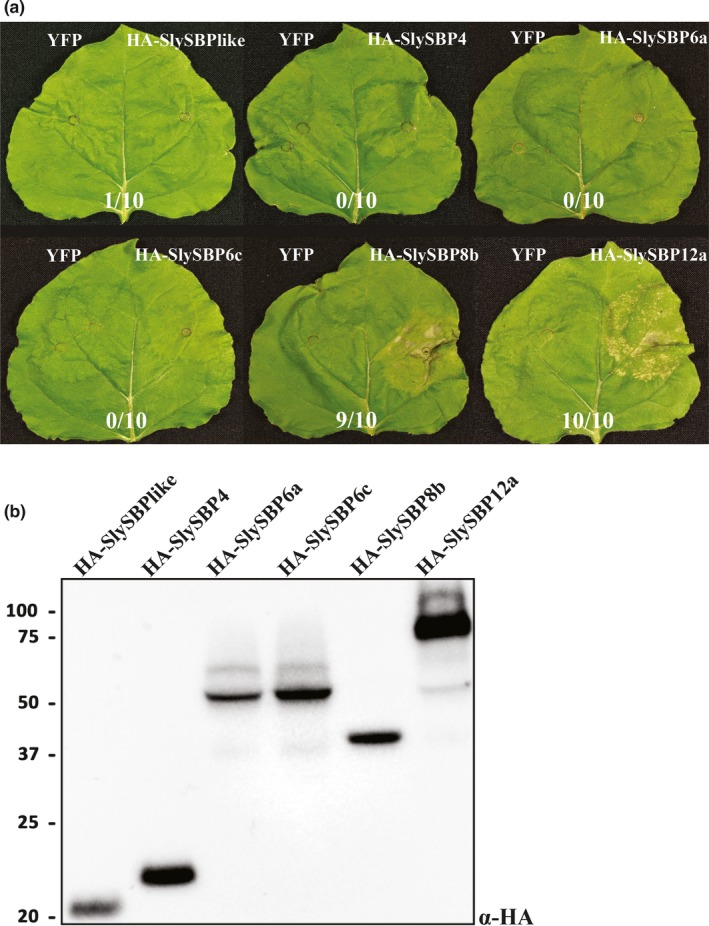
Cell death induced by overexpression of *SlySBP* transcription factors in *N. benthamiana*. Full‐length cDNA clones of enriched *SlySBP* transcription factors from the yeast two‐hybrid assay were transiently overexpressed in *N. benthamiana*. (a) The left half of each leaf was transformed with free *YFP* as a negative control while the right half was transformed with the corresponding *SlySBP* gene containing an N‐terminal HA tag and 35S promoter. Images were taken 5 days post‐transformation. Cell death was recorded if tissue collapse or lesion formation was present on the right half (*SBP*) but absent on the left half (*YFP*) of the leaf. A total of 10 leaves from five plants were scored for cell death with the results displayed below each leaf. (b) An immunoblot was performed on tissue collected 2 days post‐transformation to confirm accumulation of SlySBP proteins. Proteins were detected using an α‐HA antibody

The left half of each leaf was transformed with free *YFP* as a negative control for cell death induction while the right half was transformed with the corresponding *SBP* transcription factor. Cell death was recorded if tissue collapse or lesion formation was present on the right half (*SBP*) but absent on the left half (*YFP*) of the leaf. A total of 10 leaves from five plants were scored at 5 days post‐transformation. The results are displayed alongside a representative image of the phenotype in Figure 1a. Tissue death induced by *35S:HA‐SlySBP8b* and *35S:HA‐SlySBP12a* expression occurred in 9/10 and 10/10 leaves, respectively (Figure 1a). Overexpression of *35S:HA‐SlySBP‐like* induced weak cell death in 1/10 leaves while the other *SBP*s failed to produce any visible signs of tissue death (Figure 1a). Immunoblots using an α‐HA antibody confirmed protein accumulation for all constructs (Figure 1b). These results show that at least two SfIAP interactors induce clear signs of cell death upon overexpression in *N. benthamiana*.

### 3.3 SlySBP8b and SlySBP12a interact with SfIAP^M4^(I332A) in planta

Due to the strong tissue death phenotype associated with the overexpression of *SlySBP8b* and *SlySBP12a*, we focused our subsequent efforts on these two SBP variants. For *in vivo* confirmation of the yeast two‐hybrid results, we performed coimmunoprecipitation (CoIP) assays in *N. benthamiana* leaves. A truncated version of SfIAP beginning at the 4th methionine residue was used as our bait. This version maintains its function in *S. frugiperda* cells but lacks a caspase recognition site that is typically cleaved in *S. frugiperda* (Cerio, Vandergaast, & Friesen, 2010). This is particularly important as we show that cleavage at the N‐terminus of the full‐length protein occurs in *N. benthamiana*, thus removing N‐terminal epitope tags (Supporting information Figure S3).

We noticed that SlySBP12a was not enriched in our yeast two‐hybrid when full‐length SfIAP was used as bait but only appeared when the SfIAP_BIR1_ truncation was used (Table 1). To account for the possibility that SfIAP may interact transiently with SlySBP12a, an E3 ligase mutant of the truncated SfIAP protein was generated by mutating a conserved residue in the RING domain (Cerio et al., 2010). This construct, referred to as SfIAP^M4^(I332A), is resistant to N‐terminal cleavage in *N. benthamiana* (Supporting information Figure S3).

**Figure 2:**
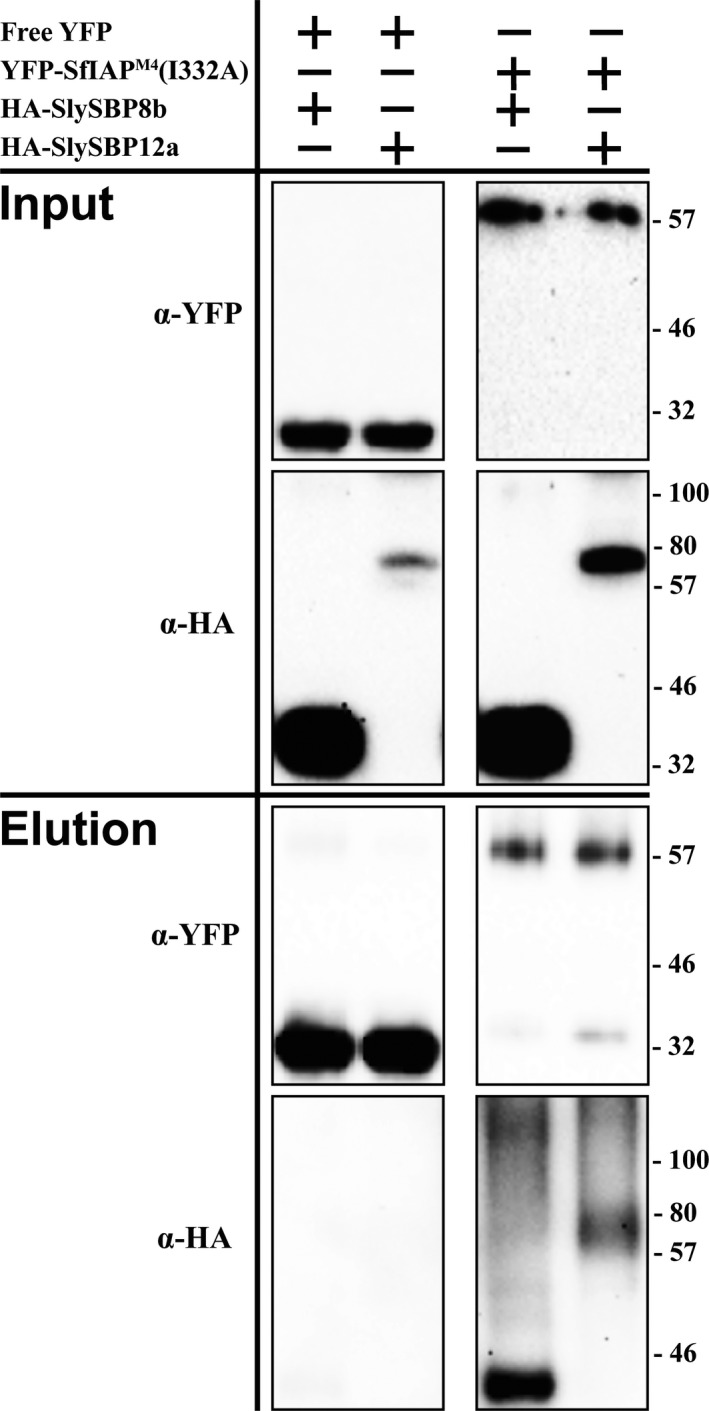
Coimmunoprecipitation of SfIAP^M^ ^4^(I332A) with SlySBP8b and SlySBP12a in *N. benthamiana*. *35S:YFP‐Sf* *IAP*^*M*^ ^*4*^ *(I332A)* or free *YFP* was transiently coexpressed with *35S:HA‐SlySBP8b* or *35S:HA‐SlySBP12a* in *N. benthamiana* leaves. Proteins were immunoprecipitated with an α‐YFP affinity matrix. A portion of each sample was taken before immunoprecipitation to serve as the input control. An immunoblot was performed on input and elution fractions using the indicated antibodies to detect the epitope‐tagged proteins

Two days after coexpression of *35S:YFP‐SfIAP* ^*M4*^ *(I332A)* with *35S:HA‐SlySBP8b* or *35S:HA‐SlySBP12a*, total proteins were extracted from leaves and incubated with GFP‐Trap_MA beads (Chromotek, Germany). All proteins were detected in the input fraction, and HA‐SlySBP8b and HA‐SlySBP12a were successfully pulled‐down by YFP‐SfIAP^M4^(I332A) but not by free YFP (Figure 2). These data confirm the yeast two‐hybrid results and demonstrate that SfIAP^M4^(I332A) interacts with SlySBP8b and SlySBP12a in plant cells.

### 3.4 SlySBP8b and SlySBP12a localize to the nucleus

**Figure 3:**
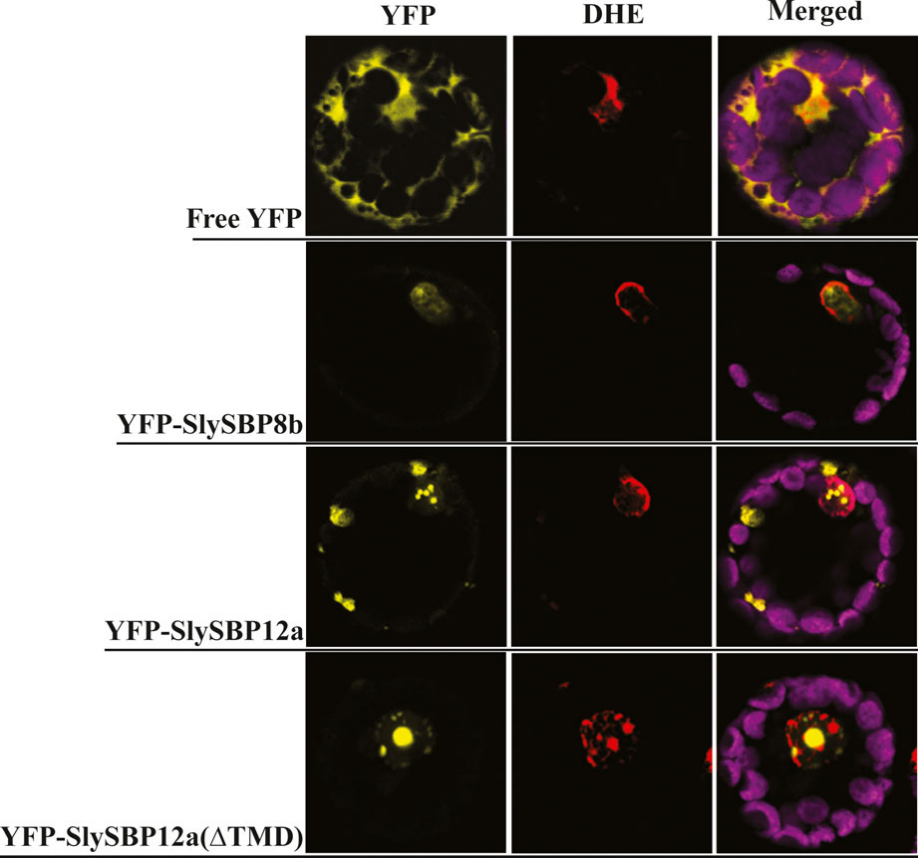
Nuclear localization of free YFP, SlySBP8b, SlySBP12a, and SlySBP12a(ΔTMD) in tomato protoplasts. Tomato protoplasts were transfected with plasmids encoding *35S:YFP*,* 35S:YFP‐SlySBP8b*,* 35S:YFP‐SlySBP12a*, or *35S:YFP‐SlySBP12a(ΔTMD)* and imaged by CLSM. Dihydroethidium (red) was used as a nuclear counterstain while the magenta signal represents chloroplast autofluorescence

As putative transcription factors, we reasoned that SlySBP8b and SlySBP12a function in the nucleus through regulation of genes involved in cell death induction. Additionally, a predicted bi‐partite nuclear localization signal (NLS) is present in the SBP domain of all tomato SBP transcription factors (Salinas, Xing, Hohmann, Berndtgen, & Huijser, 2012). Localization was assessed by expressing *35S:YFP‐SlySBP8b* and *35S:YFP‐SlySBP12a* in tomato mesophyll protoplasts. Confocal laser scanning microscopy (CLSM) revealed that both YFP‐SlySBP8b and YFP‐SlySBP12a colocalized with the nuclear marker dihydroethidium (DHE) (Figure 3).

### 3.5 Disruption of the NLS in SlySBP8b and SlySBP12a abolishes cell death induction

**Figure 4:**
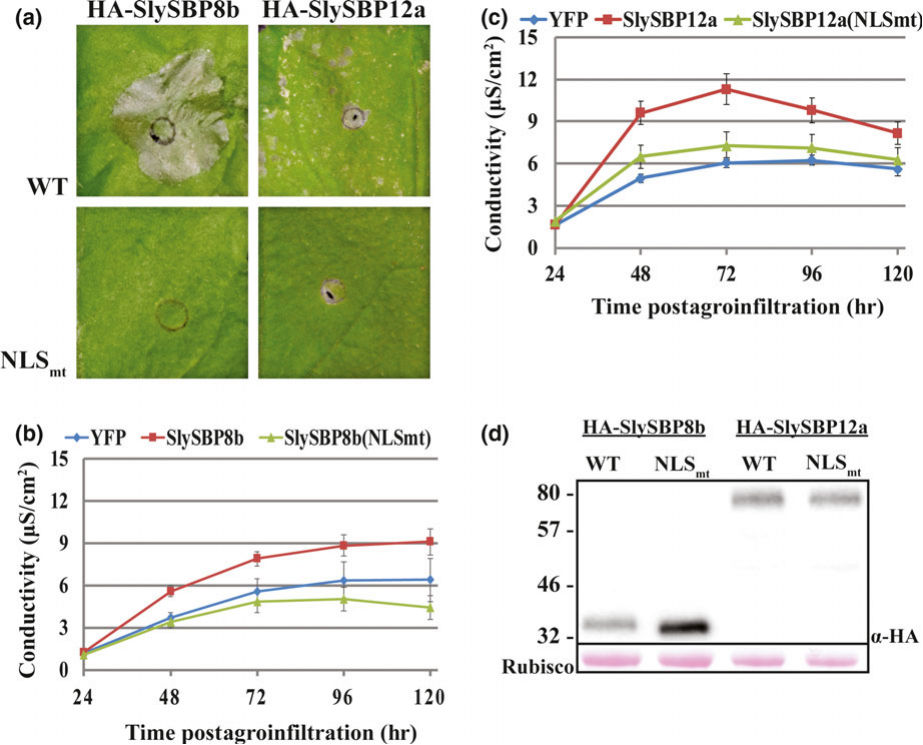
Disruption of the NLS in SlySBP8b and SlySBP12a prevents cell death in *N. benthamiana* upon overexpression. *35S:HA‐SlySBP8b, 35S:HA‐SlySBP8b(NLS*_*mt*_ *), 35S:HA‐SlySBP12a,* or *35S:HA‐SlySBP12a(NLS*_*mt*_ *)* were transiently transformed in *N. benthamiana*. (a) Images of leaves taken 5 days post‐transformation. (b) Electrolyte leakage assay used to quantify cell death. *35S:YFP*—blue diamond; *35S:HA‐SlySBP8b*—red square; *35S:HA‐SlySBP8b(NLSmt)*—green triangle. Two independent experiments were pooled together for a total of 13 biological replicates for each gene. Error bars represent a 95% confidence interval. (c) Electrolyte leakage assay used to quantify cell death. *35S:YFP*—blue diamond; *35S:HA‐SlySBP12a* —red square; *35S:HA‐SlySBP12a(NLSmt)*—green triangle. Three independent experiments were pooled together for a total of 22 biological replicates for each gene. Error bars represent a 95% confidence interval. (d) Immunoblot performed on tissue collected 2 days post‐transformation. An α‐HA antibody was used to detect SlySBP proteins, and Ponceau S stain was used to detect Rubisco as a loading control

In addition to its role in nuclear targeting, the NLS of SBP proteins forms a positively charged surface that is required for DNA binding (Birkenbihl, Jach, Saedler, & Huijser, 2005). Site‐directed mutagenesis was used to substitute conserved lysine and arginine residues in the NLS with leucine to disrupt this positive charge (Supporting information Figure S4). Overexpression of the two NLS mutants, *35S:HA‐SlySBP8b(NLS*_*mt*_ *)* and *35S:HA‐SlySBP12a(NLS*_*mt*_ *)*, in *N. benthamiana* leaves did not induce visible signs of cell death (Figure 4a). Electrolyte leakage induced by overexpression of *HA‐SlySBP8b(NLSmt)* and *HA‐SlySBP12a(NLSmt)* was similar to overexpression of the negative control *YFP* (Figure 4b and c). Immunoblots performed on tissues overexpressing both the wild‐type and NLS mutants confirmed that protein accumulation was not greatly affected by mutations in the NLS (Figure 4d).

**Figure 5:**
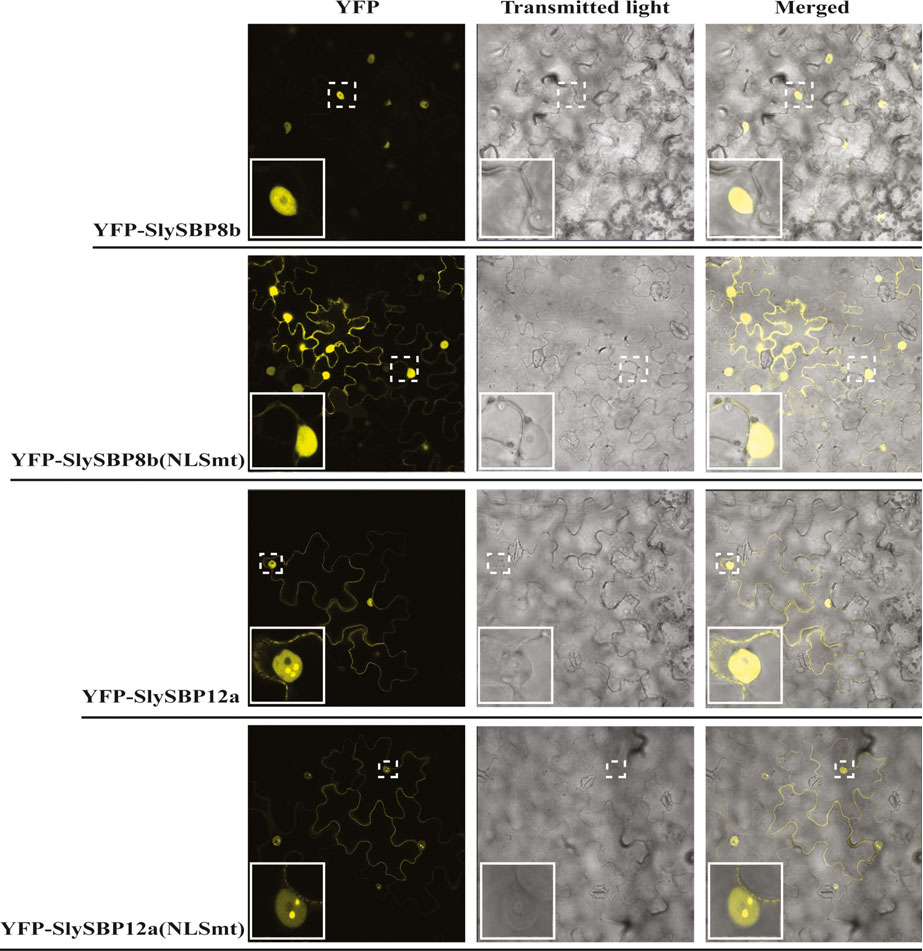
Localization of SlySBP8b, SlySBP8b(NLSmt), SlySBP12a, and SlySBP12a(NLSmt) in *N. benthamiana* epidermal cells. Leaves were transiently transformed with *35S:YFP‐SlySBP8b, 35S:YFP‐SlySBP8b(NLSmt), 35S:YFP‐SlySBP12a,* or *35S:YFP‐SlySBP12a(NLSmt)* and imaged using CLSM 2 days later. The dashed‐line box in each panel is magnified and displayed in the lower‐left corner of each panel. A transmitted light image was included to highlight the periphery of epidermal cells and nuclei

Nuclear localization of YFP‐SlySBP8b(NLSmt) and YFP‐SlySBP12a(NLSmt) in *N. benthamiana* epidermal cells was still observed (Figure 5). Localization of YFP‐SlySBP8b(NLSmt) was also observed outside of the nucleus around the periphery of the cell, which was not observed with YFP‐SlySBP8b, indicating nuclear import is impaired but not abolished in this mutant (Figure 5). The cis‐regulatory element for SBP transcription factor binding to promoters (5′‐CCGTAC(A/G)‐3′) was previously described (Franco‐Zorrilla et al., 2014; Liang, Nazarenus, & Stone, 2008). We searched for this motif in the promoter region of Arabidopsis genes and found 523 genes containing this motif within 1,000‐bp upstream of the transcription start site. These genes are involved in many diverse biological processes (Supporting information Figure S5). A closer look revealed WRKY transcription factors, NBS‐LRR genes, and an SBP homolog (Supporting information Table S2). Two of these genes, *RPP4* and *RRS1*, are known lesion‐mimic mutants in Arabidopsis (Huang, Li, Bao, Zhang, & Yang, 2010; Noutoshi et al., 2005). These data suggest a functional NLS within the SBP domain is required for cell death induction caused by *SlySBP8b* and *SlySBP12a* overexpression.

### 3.6 SlySBP12a is anchored to the ER membrane by a C‐terminal transmembrane domain

**Figure 6:**
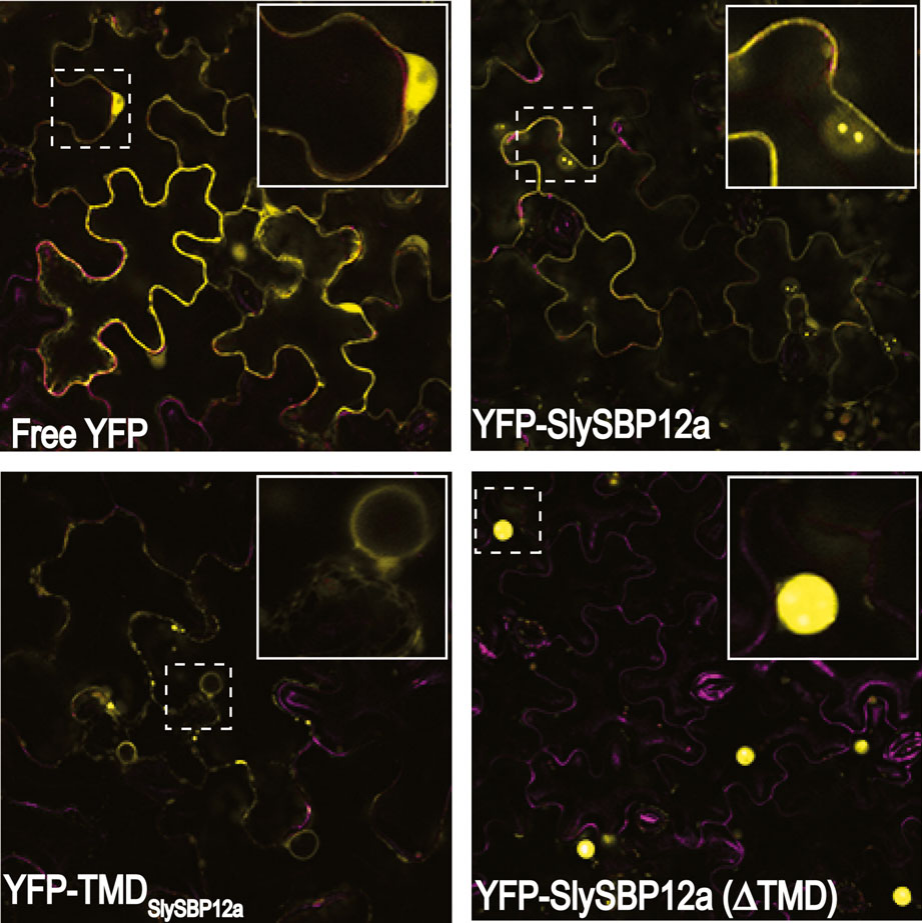
Localization of SlySBP12a, TMD_S__ly_ _SBP__12a_, and SlySBP12a(ΔTMD) in *N. benthamiana* epidermal cells. Leaves were transiently transformed with *35S:YFP‐SlySBP12a, 35S:YFP‐SlySBP12a(ΔTMD), 35S:YFP‐* *TMD*_*S*__*ly*_ _*SBP*__*12a*_ *,* or *35S:YFP* and imaged using CLSM 2 days post‐transformation. The dashed‐line box in each panel is magnified and displayed in the upper‐right corner of each panel. Chlorophyll autofluorescence is shown in magenta

**Figure 7:**
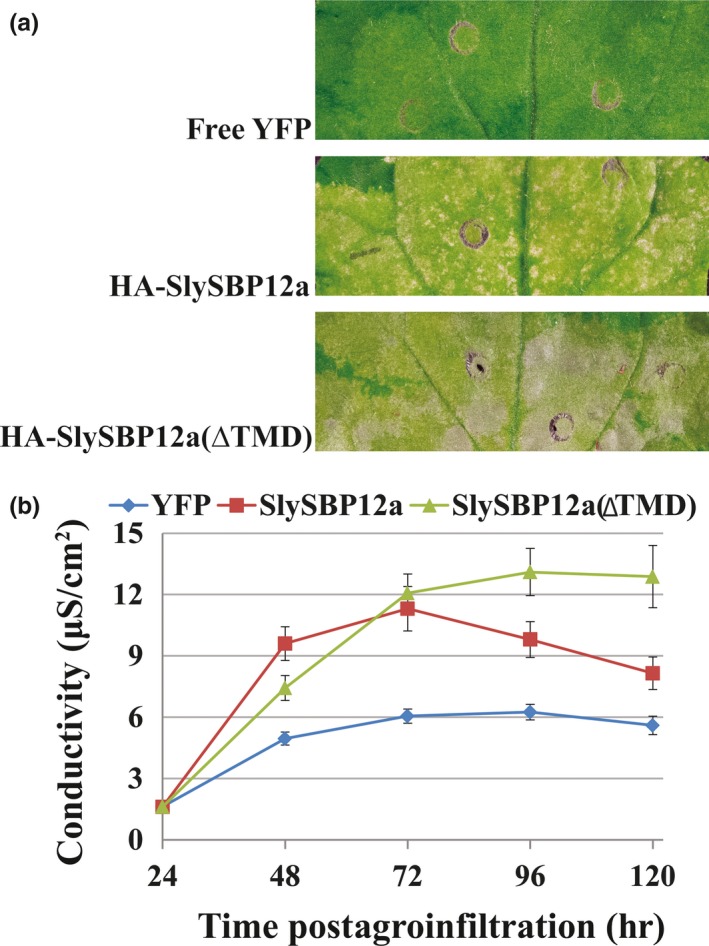
Removal of the TMD from SlySBP12a results in enhanced cell death upon overexpression. *35S:HA‐SlySBP12a, 35S:HA‐SlySBP12a(ΔTMD),* or *35S:YFP* was transiently transformed in *N. benthamiana*. (a) Images of leaves taken 5 days post‐transformation. (b) Electrolyte leakage assay used to quantify cell death. *35S:YFP*—blue diamond; *35S:HA‐SlySBP12a* —red square; and *35S:HA‐SlySBP12a(ΔTMD)*—green triangle. Three independent experiments with similar results were pooled together for a total of 22 biological replicates for each gene. Error bars represent a 95% confidence interval

While YFP‐SlySBP8b was found to be strictly nuclear localized, YFP‐SlySBP12a was also localized to diffuse pockets outside of the nucleus (Figure 3). The presence of a putative C‐terminal transmembrane domain (TMD) in SlySBP12a (Supporting information Figure S2) suggested that this localization pattern could be due to the anchoring of SlySBP12a to a cellular membrane. Removal of the last 73 amino acids of SlySBP12a eliminated the putative TMD and resulted in complete localization of YFP‐SlySBP12a(ΔTMD) to the nucleus (Figure 3; Figure 6). Additionally, overexpression of *35S:HA‐SlySBP12a(ΔTMD)* in *N. benthamiana* caused enhanced cell death characterized by extensive tissue collapse at the site of transgene expression and increased electrolyte leakage compared to the full‐length construct (Figure 7a and b). The TMD of SlySBP12a may thus regulate its access to the nucleus and the subsequent induction of cell death.

**Figure 8:**
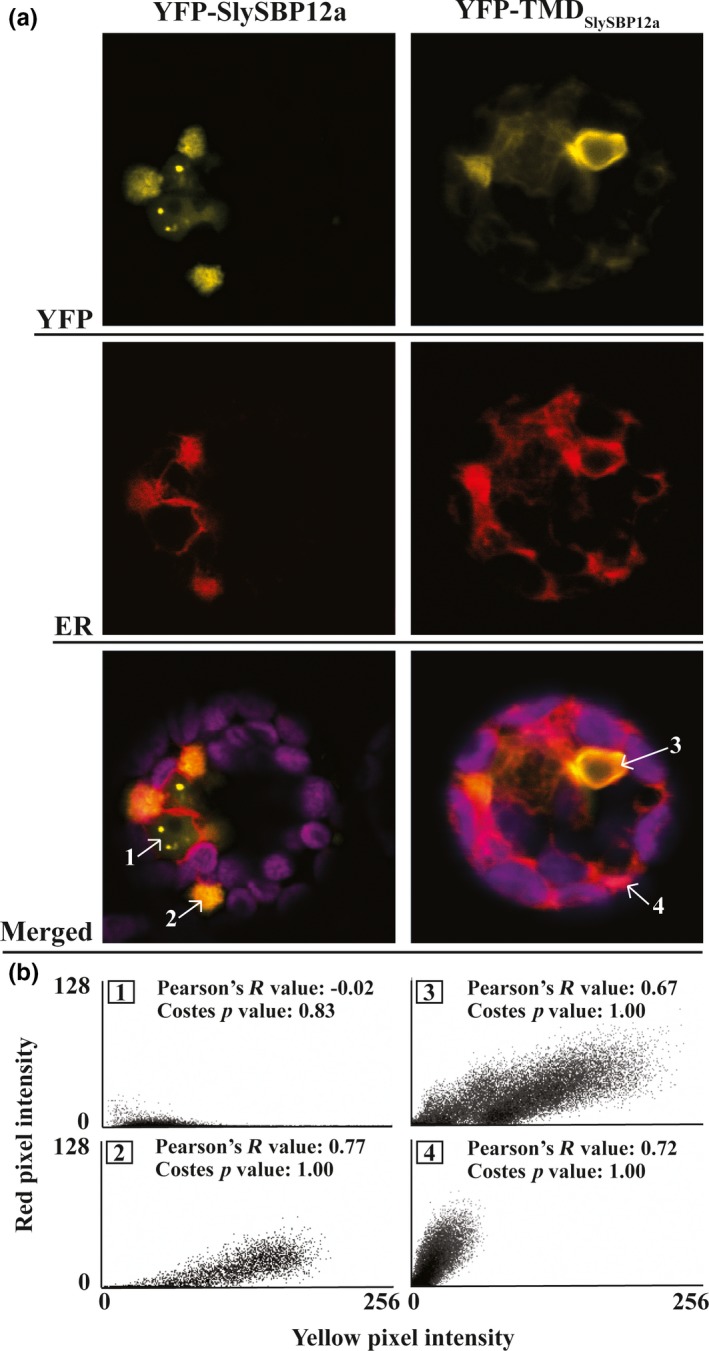
Endoplasmic reticulum localization of SlySBP12a and TMD_S__ly_ _SBP__12a_ in tomato protoplasts. Tomato protoplasts were transfected with plasmids encoding *35S:YFP‐SlySBP12a* or *35S:YFP‐* *TMD*_*S*__*ly*_ _*SBP*__*12a*_ and imaged by CLSM. An SP‐mCherry‐HDEL construct was cotransfected to serve as an ER marker (red). The magenta signal represents chloroplast autofluorescence. (a) Representative images of tomato protoplasts expressing *35S:YFP‐SlySBP12a* or *35S:* *TMD*_*S*__*ly*_ _*SBP*__*12a*_ with the ER marker. Numbered regions indicated by arrows were used for colocalization analysis. (b) Intensity histograms of the four regions selected for colocalization analysis. Pearson's *R* values and Costes *p*‐values are displayed for each region

To determine the membrane localization of SlySBP12a, the last 73 amino acids of the protein containing the putative TMD were fused to the C‐terminal end of YFP (YFP‐TMD_SlySBP12a_) (Supporting information Figure S4). This construct was expressed in *N. benthamiana* where it localized to the periphery of epidermal cells and a ring‐like structure around the nucleus that resembled endoplasmic reticulum (ER) localization (Figure 6). Endoplasmic reticulum localization was confirmed in tomato protoplasts, where the YFP‐TMD_SlySBP12a_ fusion colocalized with the ER marker SP‐mCherry‐HDEL (Figure 8a). This ER marker consists of the fluorescent protein mCherry with a signal peptide at its N‐terminus and an ER retention motif at its C‐terminus (Nelson, Cai, & Nebenfuhr, 2007). We were also able to show colocalization between the full‐length YFP‐SlySBP12a construct and the ER marker in tomato protoplasts (Figure 8a). Intensity histograms were generated, and Pearson's *R* values and Costes *p*‐values were calculated for four regions. Regions around and outside of the nucleus, where we see YFP‐SlySBP12a and YFP‐TMD_SlySBP12a_ colocalization with the ER marker, produced Pearson's *R* values of 0.77, 0.72, and 0.67 with a Costes *p*‐Value of 1.00 (Figure 8b). With Pearson's *R* values close to 1.00 and a Costes *p* value of 1.00, there is strong evidence that the YFP and mCherry signals overlap. However, inside of the nucleus where only YFP‐SlySBP12a appears to accumulate, the Pearson's *R* value was −0.02 with a Costes *p*‐value of 0.83 (Figure 8b). A Pearson's *R* value close to 0.00 and a Costes *p* value below 0.95 provide no statistically significant evidence of overlap between YFP and mCherry signals in the nucleus. Taken together, these results confirm that SlySBP12a contains a functional TMD that integrates the full‐length protein into the ER membrane.

### 3.7 ROS production and fungal growth in leaves overexpressing SlySBP8b and SlySBP12a

**Figure 9:**
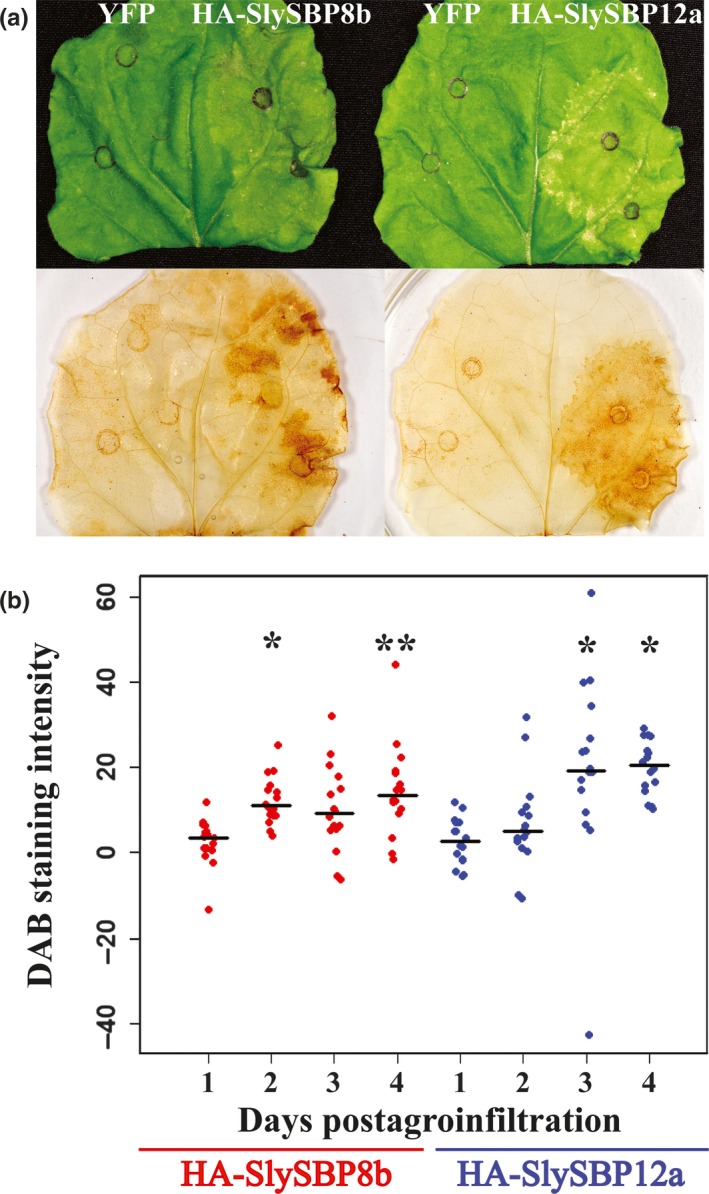
Overexpression of *SlySBP8b* and *SlySBP12a* in *N. benthamiana* induces H_2_O_2_ accumulation. *35S:HA‐SlySBP8b* and *35S:HA‐SlySBP12a* were transiently transformed in *N. benthamiana*. Leaves were cleared and stained with DAB to detect H_2_O_2_. (a) Images of leaves before and after DAB staining taken 4 days post‐agroinfiltration. (b) Quantification of DAB‐stained area for each *SlySBP* relative to *YFP* expression on the same leaf. ImageJ was used to analyze 16 leaves for each gene at each time point. Data are displayed as a dotplot with the medians represented as black horizontal lines. Statistical significance for each gene compared to its Day 1 time point was determined using a one‐way ANOVA with Tukey's HSD post hoc test (*p < 0.01; **p < 0.001)

Reactive oxygen species (ROS) are important cell death intermediaries, and their accumulation is a key feature of cell death imposed by necrotrophic fungal pathogens and the death‐inducing toxins they produce (Heller & Tudzynski, 2011; Kim, Min, & Dickman, 2008; Sakamoto, Tada, Nakayashiki, Tosa, & Mayama, 2005; Shi et al., 2007). Following transient expression in *N. benthamiana* leaves, we monitored the accumulation of hydrogen peroxide (H_2_O_2_) for 4 days using 3′3‐diaminobenzidine (DAB) staining. Leaves expressing *35S*:*HA‐SlySBP8b* and *35S:HA‐SlySBP12a* displayed enhanced DAB staining intensity relative to expression of the *35S:YFP* control on the same leaf (Figure 9a). Accumulation of H_2_O_2_ occurred as early as 2 and 3 days post‐transformation for *35S:HA‐SlySBP8b* and *35S:HA‐SlySBP12a*, respectively (Figure 9b). ImageJ software was used to measure DAB staining intensity (Schindelin et al., 2012).

Transgenic *SfIAP* plants were reported to accumulate lower levels of ROS under stress conditions compared to wild‐type plants (Li et al., 2010). Necrotrophic fungal pathogens are known to exploit host ROS production as means to kill host cells for their own benefit (Govrin & Levine, 2000; Heller & Tudzynski, 2011; Kabbage et al., 2013; Ranjan et al., 2017). In addition to reduced ROS accumulation, transgenic *SfIAP* plants are also resistant to the necrotrophic fungal pathogen *A. alternata* (Li et al., 2010). Therefore, we reasoned that leaves overexpressing *SlySBP8b* and *SlySBP12a* would support enhanced growth of this pathogen. Unfortunately, *N. benthamiana* is not susceptible to this pathogen, so we screened *Nicotiana* germplasm for susceptible species. We found that *Nicotiana glutinosa* was susceptible to *A. alternata* and previous work confirmed that transgenes could be expressed effectively in this species using Agrobacterium‐mediated transient transformation (Kessens, Ashfield, Kim, & Innes, 2014).

**Figure 10:**
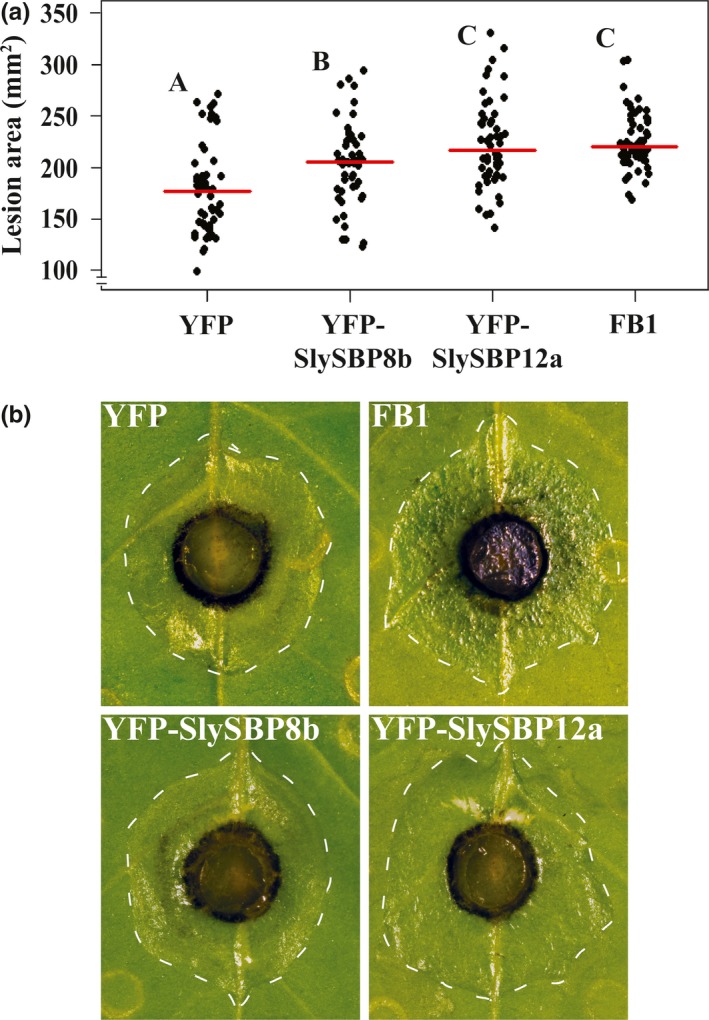
Overexpression of *SlySBP8b* and *SlySBP12a* enhances *A. alternata* growth on *N. glutinosa*. *35S:YFP‐SlySBP8b, 35S:YFP‐SlySBP12a,* or *35S:YFP* was transiently transformed in *N. glutinosa*. As a positive control for cell death induction, leaves were treated with 5 μM FB1. Agar plugs containing actively growing *A. alternata* mycelium were placed fungal‐side‐down on leaves. Images were taken, and lesion areas were recorded, 3 days after fungal inoculation. (a) Quantification of lesion area using ImageJ. The results of four randomized and blind experiments were pooled representing 54 leaves for each treatment. Data are displayed as a dotplot with the medians represented by red horizontal lines. Treatments with the same letter are not statistically significant as determined by a one‐way ANOVA with Tukey's HSD post hoc test (YFP/SBP8b, *p* = 0.02; YFP/SBP12a, *p* = 2.0E‐7; YFP/FB1, *p* = 5.0 E‐7; SBP8b/SBP12a, *p* = 0.02; SBP8b/FB1, *p* = 0.04). (b) Images of inoculated leaves with lesions outlined by a dotted white line

While the differences were small, a total of 54 biological replicates from four randomized and blind experiments showed that leaves expressing *35S:YFP‐SlySBP8b* or *35S:YFP‐SlySBP12a* had increased *A. alternata* lesion areas compared to leaves expressing *35S:YFP* alone (Figure 10a and b). This effect was more pronounced with *35S:YFP‐SlySBP12a* than with *35S:YFP‐SlySBP8b* expression. As a positive control, leaves were treated with 5 μM FB1 to simulate cell death induction by a fungal toxin. Lesion development in *35S:YFP‐SlySBP12a*‐expressing tissue and FB1‐treated tissue was comparable (Figure 10b). Fluorescence microscopy was used to confirm protein accumulation in each leaf before fungal inoculation, and *35S:YFP‐SlySBP8b* and *35S:YFP‐SlySBP12a* were able to induce tissue death in *N. glutinosa* (Supporting information Figure S6). Lesion areas were measured using ImageJ software (Schindelin et al., 2012). Overall, we show that these two transcription factors are able to increase ROS levels and promote *A. alternata* growth, phenotypes that are dampened in plants expressing *SfIAP*.

## 4 DISCUSSION

Over 15 years ago, it was first reported that heterologous expression of a viral IAP (*OpIAP*) in tobacco suppressed cell death induced by the necrotrophic fungal pathogen *S. sclerotiorum* and the necrosis‐inducing viral pathogen tomato spotted wilt virus (Dickman et al., 2001). Subsequent studies revealed that an IAP from *Spodoptera frugiperda* (SfIAP) suppressed cell death imposed by numerous abiotic and biotic stresses (Hoang, Williams, Khanna, Dale, & Mundree, 2014; Kabbage et al., 2010; Li et al., 2010). However, the biochemical mechanism by which these IAPs suppress cell death in plant systems remains unknown. In this study, we utilize SfIAP as a biochemical tool to identify novel prodeath regulators and shed light on how SfIAP functions in plants.

### 4.1 SlySBP8b and SlySBP12a associate with SfIAP^M4^(I332A) and exhibit characteristics of prodeath regulators

The yeast two‐hybrid and CoIP data presented clearly show that SlySBP8b and SlySBP12a associate with SfIAP^M4^(I332A) (Table 1 & Figure 2). Remarkably, *SlySBP8b* and *SlySBP12a* exhibit attributes of prodeath regulators, demonstrated by cell death induction and ROS accumulation upon overexpression (Figures 1 and 9). We anticipated that coexpression of *SfIAP* with *SlySBP8b* or *SlySBP12a* would suppress cell death induction. However, numerous attempts to suppress cell death induced by *SlySBP8b* and *SlySBP12a* overexpression were unsuccessful when *SfIAP* or *SfIAP* ^*M4*^ was coexpressed. One possible explanation is the fact that SfIAP and SfIAP^M4^ protein accumulation is significantly less than SlySBP8b and SlySBP12a when expressed in *N. benthamiana*. In fact, SfIAP and SfIAP^M4^ accumulation was only detected when these proteins were fused to YFP. While an N‐terminal YFP tag did not impact binding to SlySBP8b and SlySBP12a, we cannot rule out the possibility that this large tag could affect the ability of SfIAP or SfIAP^M4^ to suppress the activity of these transcription factors. An excess of either transcription factor could allow enough to enter the nucleus and influence cell death gene expression.

SlySBP8b and SlySBP12a belong to a family of plant‐specific transcription factors known as SQUAMOSA promoter‐binding proteins (SBPs), of which 15 members are present in tomato (Salinas et al., 2012). Members of this family are defined by a highly conserved SBP box DNA binding domain and can be further divided into nine phylogenetically distinct clades (Preston & Hileman, 2013; Yamasaki, Kigawa, Seki, Shinozaki, & Yokoyama, 2013). *SBP* genes are known to regulate diverse developmental processes such as flowering time, branching, trichome development, apical dominance, and pollen sac development to name a few (Wang & Wang, 2015; Yamasaki et al., 2013). Interestingly, silencing of the *SBP* gene *Colorless non‐ripening* (*Cnr*) in tomato results in fruit with delayed ripening, a phenotype observed in tomatoes overexpressing *SfIAP* (Li et al., 2010; Manning et al., 2006).

While much is known about the role of SBP transcription factors in plant development, only a few studies to date have associated SBPs with stress responses. The deletion of Arabidopsis *SPL14* (*AtSPL14*) conferred enhanced tolerance to FB1, thus implicating this SBP transcription factor in the cellular response to this mycotoxin (Stone, Liang, Nekl, & Stiers, 2005). Tolerance to FB1 is a phenotype that we have also observed in *SfIAP‐*overexpressing tomato seedlings (Li et al., 2010). Interestingly, AtSPL14 and SlySBP12a both reside in clade‐II and display similar structural characteristics with large SBP proteins that contain a predicted C‐terminal transmembrane domain (Preston & Hileman, 2013).

Another clade‐II member, *GmSPL12l* from soybean, was shown to be a target of the *Phakopsora pachyrhizi* (Asian soybean rust) effector PpEC23 (Qi et al., 2016). This effector suppressed the hypersensitive response (HR) in soybean and tobacco and also interacted with other clade‐II members from *N. benthamiana* and Arabidopsis: NbSPL1 and AtSPL1 (Qi et al., 2016). In another study, the N immune receptor of *N. benthamiana* was found to associate with the SBP transcription factor NbSPL6 upon activation of HR. This interaction only occurred when plants were challenged with an HR‐eliciting strain of tobacco mosaic virus (TMV‐U1) but not a non‐eliciting strain (TMV‐Ob) (Padmanabhan et al., 2013). Taken together, our results and the findings of previous studies clearly show that SBP transcription factors are critical regulators of plant stress responses that result in cell death.

Fungal pathogens with a necrotrophic lifestyle are known to exploit host ROS production for cell death induction and successful pathogenesis (Govrin & Levine, 2000; Heller & Tudzynski, 2011). As positive regulators of cell death and ROS production, we hypothesized that overexpression of *SlySBP8b* and *SlySBP12a* would support enhanced growth of necrotrophic fungal pathogens. Additionally, *SfIAP* transgenic plants are resistant to cell death induced by the necrotrophic fungal pathogen *A. alternata* (Li et al., 2010). The results of four randomized and blind experiments clearly show that while the contribution of *SlySBP8b* or *SlySBP12a* overexpression to *A. alternata* lesion areas was small, it was significantly greater than leaves expressing the negative control *35S:YFP* (Figure 10). The small differences in growth could be explained by the fact that *A. alternata* is already an aggressive pathogen and the benefits of priming its host for death would be small. To test this, we also treated leaves with FB1, which is a structural analog of the AAL toxin produced by *A. alternata* f. sp. *lycopersici* that induces cell death in tomato (Mirocha et al., 1992). Pre‐treatment of *N. glutinosa* leaves with FB1 led to enhanced growth of *A. alternata* comparable to *SlySBP12a* overexpression (Figure 10). These results provide further evidence that SlySBP8b and SlySBP12a are positive regulators of cell death, which in this case, contribute to pathogenic development of *A. alternata*.

### 4.2 SlySBP8b and SlySBP12a require a functional SBP domain for cell death induction

As members of a transcription factor family, we hypothesize that SlySBP8b and SlySBP12a exert their prodeath activity through the regulation of genes involved in cell death. The NLS of SBP transcription factors serves a dual role in nuclear import and DNA binding, making this an essential motif for SBP function (Birkenbihl et al., 2005). We show that these transcription factors are clearly localized to the nucleus of tomato protoplasts and *N. benthamiana* epidermal cells (Figures 3 and 5) and mutation of the bi‐partite NLS of both transcription factors abolishes cell death (Figure 4). While the NLS mutation was unable to abolish nuclear localization of SlySBP8b(NLSmt) or SlySBP12a(NLSmt), it did result in accumulation of some SlySBP8b(NLSmt) protein in the cytoplasm, likely due to partial impairment of nuclear import (Figure 5). These results highlight the importance of a functional SBP domain for cell death induction caused by *SlySBP8b* and *SlySBP12a* overexpression, possibly through the regulation of genes involved in cell death.

To identify genes involved in cell death that may be regulated by SBP transcription factors, we searched the Arabidopsis genome for promoters that contain the SBP cis‐element (Supporting information Figure S5). We identified 523 genes involved in a diverse array of biological processes, which is expected of a large transcription factor family known to be involved in diverse developmental and stress‐related processes. Further investigation of these genes revealed a subset with known roles in stress responses (Supporting information Table S2). Four genes encode nucleotide‐binding site leucine‐rich repeat (NBS‐LRR) proteins, which are known to play important roles in plant immunity through activation of hypersensitive‐programmed cell death (HR‐PCD) (McHale, Tan, Koehl, & Michelmore, 2006). Additionally, several WRKY transcription factors with known regulatory roles in plant immunity to both biotrophic and necrotrophic pathogens were identified (Bhattarai, Atamian, Kaloshian, & Eulgem, 2010; Hsu et al., 2013; Lai, Vinod, Zheng, Fan, & Chen, 2008). Perhaps the most interesting finding is that two genes, *RPP4* and *RRS1*, are known lesion‐mimic mutants (Huang et al., 2010; Noutoshi et al., 2005). Due to the large number of genes with predicted SBP‐binding sites in their promoters, future studies will need to utilize chromatin immunoprecipitation sequencing (ChIP‐Seq) to determine genes regulated by SlySBP8b and SlySBP12a *in vivo*. These studies will provide more concrete information on the downstream components responsible for cell death execution in plants.

### 4.3 SlySBP12a localizes to the ER

Unlike SlySBP8b, which we found to be strictly nuclear localized, SlySBP12a was also present outside of the nucleus (Figures 3 and 6). By fusing the putative C‐terminal TMD of SlySBP12a to YFP, we were able to show that the TMD of SlySBP12a localized YFP around the nucleus and at the periphery of *N. benthamiana* epidermal cells (Figure 6). We hypothesized that this pattern was due to ER localization. This was confirmed in tomato protoplasts, where both YFP‐SlySBP12a and YFP‐TMD_SlySBP12a_ colocalize with the ER marker SP‐mCherry‐HDEL (Figure 8a and b).

In response to environmental stress, plant cells increase production of secreted proteins, which in turn can cause ER stress due to the sudden influx of proteins that must be properly folded before moving through the rest of the secretory pathway (Eichmann & Schafer, 2012). This makes the ER an important sensor of cellular stress as the accumulation of unfolded proteins is first detected by the ER. Membrane‐tethered transcription factors (MTTFs) residing at the ER membrane play important roles in ER stress perception and regulation of genes involved in stress relief and cell death in mammalian and plant systems (Slabaugh & Brandizzi, 2011). Membrane tethering provides spatial regulation of transcription factor activity, as MTTFs must be removed from the membrane before the transcription factor domain can translocate to the nucleus (Slabaugh & Brandizzi, 2011). This type of regulation allows these transcription factors to act quickly in response to cellular stress.

In this study, we show that SlySBP12a exhibits a localization pattern similar to previously described ER‐MTTFs from Arabidopsis: NAC089, bZIP28, and bZIP60. These transcription factors are activated upon perception of ER stress and activate cell death (NAC089), heat stress (bZIP28), and ER stress (bZIP60) responses through transcriptional regulation of genes involved in these processes (Gao, Brandizzi, Benning, & Larkin, 2008; Iwata & Koizumi, 2005; Liu, Srivastava, Che, & Howell, 2007; Yang et al., 2014). Removal of the TMD from these transcription factors results in their complete localization to the nucleus and constitutive activation of the processes they regulate (Gao et al., 2008; Iwata & Koizumi, 2005; Liu et al., 2007; Yang et al., 2014). This mirrors what we have observed with SlySBP12a. Removal of the TMD results in complete nuclear localization in *N. benthamiana* and tomato cells and enhanced cell death induction compared to full‐length SlySBP12a (Figures 3, 6, and 7).

With our data and previous studies of ER‐MTTFs, we can speculate that SlySBP12a is cleaved from the ER membrane upon stress perception and translocates to the nucleus where it regulates genes involved in cell death. However, we must keep in mind that our experiments were performed with a cDNA copy of SlySBP12a, preventing the detection of splice isoforms that could lack the TMD. This is important to consider as bZIP60 was originally thought to be proteolytically cleaved from the ER membrane upon stress induced by tunicamycin treatment (Iwata, Fedoroff, & Koizumi, 2008). A follow‐up study by the same group showed that in addition to being proteolytically cleaved, bZIP60 is also alternatively spliced in response to tunicamycin treatment, resulting in a truncated protein lacking the C‐terminal TMD (Nagashima et al., 2011). Future experiments looking at the translocation of SlySBP12a upon stress induction must consider the possibility of alternative splice isoforms.

## 5 CONCLUSION

While the expression of *IAP* and other anti‐apoptotic genes in plants confers enhanced stress tolerance, the animal‐derived nature of these genes will likely prevent their broad commercial use. Thus, the identification of endogenous plant cell death regulators, such as SBP transcription factors, that can be targeted to ameliorate stress tolerance is appealing. This is exemplified by recent interest in exploiting *SBP* genes for crop improvement due to the many developmental traits they regulate (Liu, Harberd Nicholas, & Fu, 2016; Wang & Wang, 2015). Efforts are underway in our laboratory to determine whether the disruption of these transcription factors impact tolerance to a range of abiotic and biotic insults.

## AUTHOR CONTRIBUTIONS

R.K. performed and designed experiments, analyzed data, and wrote the manuscript. N.S. performed experiments and analyzed data. M.K. performed and designed experiments, analyzed data, and wrote the manuscript. All authors reviewed the manuscript.

## References

[pld381-bib-0001] Afgan, E. , Baker, D. , van den Beek, M. , Blankenberg, D. , Bouvier, D. , Cech, M. , … Goecks, J. (2016). The Galaxy platform for accessible, reproducible and collaborative biomedical analyses: 2016 update. Nucleic Acids Research, 44, W3–W10. 27137889 10.1093/nar/gkw343 PMC4987906

[pld381-bib-0002] Allocati, N. , Masulli, M. , Di Ilio, C. , & De Laurenzi, V. (2015). Die for the community: An overview of programmed cell death in bacteria. Cell Death & Disease, 6, e1609. 25611384 10.1038/cddis.2014.570 PMC4669768

[pld381-bib-0003] Asai, T. , Stone, J. M. , Heard, J. E. , Kovtun, Y. , Yorgey, P. , Sheen, J. , & Ausubel, F. M. (2000). Fumonisin B1‐induced cell death in arabidopsis protoplasts requires jasmonate‐, ethylene‐, and salicylate‐dependent signaling pathways. Plant Cell, 12, 1823–1836. 11041879 10.1105/tpc.12.10.1823 PMC149122

[pld381-bib-0004] Bhattarai, K. K. , Atamian, H. S. , Kaloshian, I. , & Eulgem, T. (2010). WRKY72‐type transcription factors contribute to basal immunity in tomato and Arabidopsis as well as gene‐for‐gene resistance mediated by the tomato R gene Mi‐1. The Plant Journal, 63, 229–240. 20409007 10.1111/j.1365-313X.2010.04232.x

[pld381-bib-0005] Birkenbihl, R. P. , Jach, G. , Saedler, H. , & Huijser, P. (2005). Functional dissection of the plant‐specific SBP‐domain: Overlap of the DNA‐binding and nuclear localization domains. Journal of Molecular Biology, 352, 585–596. 16095614 10.1016/j.jmb.2005.07.013

[pld381-bib-0006] Bolger, A. M. , Lohse, M. , & Usadel, B. (2014). Trimmomatic: A flexible trimmer for Illumina sequence data. Bioinformatics, 30, 2114–2120. 24695404 10.1093/bioinformatics/btu170 PMC4103590

[pld381-bib-0007] Cerio, R. J. , Vandergaast, R. , & Friesen, P. D. (2010). Host insect inhibitor‐of‐apoptosis SfIAP functionally replaces baculovirus IAP but is differentially regulated by Its N‐terminal leader. Journal of Virology, 84, 11448–11460. 20739517 10.1128/JVI.01311-10 PMC2953141

[pld381-bib-0008] Dickman, M. B. , Park, Y. K. , Oltersdorf, T. , Li, W. , Clemente, T. , & French, R. (2001). Abrogation of disease development in plants expressing animal antiapoptotic genes. Proceedings of the National Academy of Sciences of the United States of America, 98, 6957–6962. 11381106 10.1073/pnas.091108998 PMC34460

[pld381-bib-0009] Earley, K. W. , Haag, J. R. , Pontes, O. , Opper, K. , Juehne, T. , Song, K. , & Pikaard, C. S. (2006). Gateway‐compatible vectors for plant functional genomics and proteomics. The Plant Journal, 45, 616–629. 16441352 10.1111/j.1365-313X.2005.02617.x

[pld381-bib-0010] Eichmann, R. , & Schafer, P. (2012). The endoplasmic reticulum in plant immunity and cell death. Frontiers in Plant Science, 3, 200. 22936941 10.3389/fpls.2012.00200 PMC3424470

[pld381-bib-0011] Feltham, R. , Khan, N. , & Silke, J. (2012). IAPS and ubiquitylation. IUBMB Life, 64, 411–418. 22362579 10.1002/iub.565

[pld381-bib-0012] Franco‐Zorrilla, J. M. , López‐Vidriero, I. , Carrasco, J. L. , Godoy, M. , Vera, P. , & Solano, R. (2014). DNA‐binding specificities of plant transcription factors and their potential to define target genes. Proceedings of the National Academy of Sciences, 111, 2367–2372. 10.1073/pnas.1316278111 PMC3926073 24477691

[pld381-bib-0013] Fuchs, Y. , & Steller, H. (2011). Programmed cell death in animal development and disease. Cell, 147, 742–758. 22078876 10.1016/j.cell.2011.10.033 PMC4511103

[pld381-bib-0014] Gao, H. , Brandizzi, F. , Benning, C. , & Larkin, R. M. (2008). A membrane‐tethered transcription factor defines a branch of the heat stress response in Arabidopsis thaliana. Proceedings of the National Academy of Sciences of the United States of America, 105, 16398–16403. 18849477 10.1073/pnas.0808463105 PMC2571009

[pld381-bib-0015] Gilchrist, D. G. (1997). Mycotoxins reveal connections between plants and animals in apoptosis and ceramide signaling. Cell Death and Differentiation, 4, 689–698. 16465281 10.1038/sj.cdd.4400312

[pld381-bib-0016] Glenn, A. E. , Zitomer, N. C. , Zimeri, A. M. , Williams, L. D. , Riley, R. T. , & Proctor, R. H. (2008). Transformation‐mediated complementation of a FUM gene cluster deletion in Fusarium verticillioides restores both fumonisin production and pathogenicity on maize seedlings. Molecular Plant‐Microbe Interactions, 21, 87–97. 18052886 10.1094/MPMI-21-1-0087

[pld381-bib-0017] Govrin, E. M. , & Levine, A. (2000). The hypersensitive response facilitates plant infection by the necrotrophic pathogen Botrytis cinerea. Current Biology, 10, 751–757. 10898976 10.1016/s0960-9822(00)00560-1

[pld381-bib-0018] Gyrd‐Hansen, M. , & Meier, P. (2010). IAPs: From caspase inhibitors to modulators of NF‐kappaB, inflammation and cancer. Nature Reviews Cancer, 10, 561–574. 20651737 10.1038/nrc2889

[pld381-bib-0019] Heller, J. , & Tudzynski, P. (2011). Reactive oxygen species in phytopathogenic fungi: Signaling, development, and disease. Annual Review of Phytopathology, 49, 369–390. 10.1146/annurev-phyto-072910-095355 21568704

[pld381-bib-0020] Hoang, T. M. L. , Williams, B. , Khanna, H. , Dale, J. , & Mundree, S. G. (2014). Physiological basis of salt stress tolerance in rice expressing the antiapoptotic gene SfIAP. Functional Plant Biology, 41, 1168. 10.1071/FP13308 32481066

[pld381-bib-0021] Hsu, F. C. , Chou, M. Y. , Chou, S. J. , Li, Y. R. , Peng, H. P. , & Shih, M. C. (2013). Submergence confers immunity mediated by the WRKY22 transcription factor in Arabidopsis. Plant Cell, 25, 2699–2713. 23897923 10.1105/tpc.113.114447 PMC3753392

[pld381-bib-0022] Huang, X. , Li, J. , Bao, F. , Zhang, X. , & Yang, S. (2010). A gain‐of‐function mutation in the Arabidopsis disease resistance gene RPP4 confers sensitivity to low temperature. Plant Physiology, 154, 796–809. 20699401 10.1104/pp.110.157610 PMC2949010

[pld381-bib-0023] Iwata, Y. , Fedoroff, N. V. , & Koizumi, N. (2008). Arabidopsis bZIP60 is a proteolysis‐activated transcription factor involved in the endoplasmic reticulum stress response. Plant Cell, 20, 3107–3121. 19017746 10.1105/tpc.108.061002 PMC2613661

[pld381-bib-0024] Iwata, Y. , & Koizumi, N. (2005). An Arabidopsis transcription factor, AtbZIP60, regulates the endoplasmic reticulum stress response in a manner unique to plants. Proceedings of the National Academy of Sciences of the United States of America, 102, 5280–5285. 15781873 10.1073/pnas.0408941102 PMC555978

[pld381-bib-0025] Kabbage, M. , Kessens, R. , Bartholomay, L. C. , & Williams, B. (2017). The life and death of a plant cell. Annual Review of Plant Biology, 68, 375–404. 10.1146/annurev-arplant-043015-111655 28125285

[pld381-bib-0026] Kabbage, M. , Li, W. , Chen, S. , & Dickman, M. B. (2010). The E3 ubiquitin ligase activity of an insect anti‐apoptotic gene (SfIAP) is required for plant stress tolerance. Physiological and Molecular Plant Pathology, 74, 351–362.

[pld381-bib-0027] Kabbage, M. , Williams, B. , & Dickman, M. B. (2013). Cell death control: The interplay of apoptosis and autophagy in the pathogenicity of Sclerotinia sclerotiorum. PLOS Pathogens, 9, e1003287. 23592997 10.1371/journal.ppat.1003287 PMC3623803

[pld381-bib-0028] Kessens, R. , Ashfield, T. , Kim, S. H. , & Innes, R. W. (2014). Determining the GmRIN4 requirements of the soybean disease resistance proteins Rpg1b and Rpg1r using a nicotiana glutinosa‐based agroinfiltration system. PLoS ONE, 9, e108159. 25244054 10.1371/journal.pone.0108159 PMC4171518

[pld381-bib-0029] Kim, K. S. , Min, J. Y. , & Dickman, M. B. (2008). Oxalic acid is an elicitor of plant programmed cell death during Sclerotinia sclerotiorum disease development. Molecular Plant‐Microbe Interactions, 21, 605–612. 18393620 10.1094/MPMI-21-5-0605

[pld381-bib-0030] Kroemer, G. , Galluzzi, L. , Vandenabeele, P. , Abrams, J. , Alnemri, E. S. , Baehrecke, E. H. , … Melino, G. (2009). Classification of cell death: Recommendations of the Nomenclature Committee on Cell Death 2009. Cell Death and Differentiation, 16, 3–11. 18846107 10.1038/cdd.2008.150 PMC2744427

[pld381-bib-0031] Lacomme, C. , & Santa, Cruz. S. (1999). Bax‐induced cell death in tobacco is similar to the hypersensitive response. Proceedings of the National Academy of Sciences of the United States of America, 96, 7956–7961. 10393929 10.1073/pnas.96.14.7956 PMC22169

[pld381-bib-0032] Lai, Z. , Vinod, K. , Zheng, Z. , Fan, B. , & Chen, Z. (2008). Roles of Arabidopsis WRKY3 and WRKY4 transcription factors in plant responses to pathogens. BMC Plant Biology, 8, 68. 18570649 10.1186/1471-2229-8-68 PMC2464603

[pld381-bib-0033] Langmead, B. , & Salzberg, S. L. (2012). Fast gapped‐read alignment with Bowtie 2. Nature Methods, 9, 357–359. 22388286 10.1038/nmeth.1923 PMC3322381

[pld381-bib-0034] Lewis, J. D. , Wan, J. , Ford, R. , Gong, Y. , Fung, P. , Nahal, H. , … Guttman, D. S. (2012). Quantitative interactor screening with next‐generation sequencing (QIS‐Seq) identifies Arabidopsis thaliana MLO2 as a target of the Pseudomonas syringae type III effector HopZ2. BMC Genomics, 13, 8. 22230763 10.1186/1471-2164-13-8 PMC3320541

[pld381-bib-0035] Li, W. , Kabbage, M. , & Dickman, M. B. (2010). Transgenic expression of an insect inhibitor of apoptosis gene, SfIAP, confers abiotic and biotic stress tolerance and delays tomato fruit ripening. Physiological and Molecular Plant Pathology, 74, 363–375.

[pld381-bib-0036] Liang, X. , Nazarenus, T. J. , & Stone, J. M. (2008). Identification of a consensus DNA‐binding site for the Arabidopsis thaliana SBP domain transcription factor, AtSPL14, and binding kinetics by surface plasmon resonance. Biochemistry, 47, 3645–3653. 18302343 10.1021/bi701431y

[pld381-bib-0037] Liu, Q. , Harberd Nicholas, P. , & Fu, X. (2016). SQUAMOSA promoter binding protein‐like transcription factors: Targets for improving cereal grain yield. Molecular Plant, 9, 765–767. 27108382 10.1016/j.molp.2016.04.008

[pld381-bib-0038] Liu, J. X. , Srivastava, R. , Che, P. , & Howell, S. H. (2007). An endoplasmic reticulum stress response in Arabidopsis is mediated by proteolytic processing and nuclear relocation of a membrane‐associated transcription factor, bZIP28. Plant Cell, 19, 4111–4119. 18156219 10.1105/tpc.106.050021 PMC2217655

[pld381-bib-0039] Lorang, J. , Kidarsa, T. , Bradford, C. S. , Gilbert, B. , Curtis, M. , Tzeng, S. C. , … Wolpert, T. J. (2012). Tricking the guard: Exploiting plant defense for disease susceptibility. Science, 338, 659–662. 23087001 10.1126/science.1226743 PMC4125361

[pld381-bib-0040] Manning, K. , Tor, M. , Poole, M. , Hong, Y. , Thompson, A. J. , King, G. J. , … Seymour, G. B. (2006). A naturally occurring epigenetic mutation in a gene encoding an SBP‐box transcription factor inhibits tomato fruit ripening. Nature Genetics, 38, 948–952. 16832354 10.1038/ng1841

[pld381-bib-0041] McHale, L. , Tan, X. , Koehl, P. , & Michelmore, R. W. (2006). Plant NBS‐LRR proteins: Adaptable guards. Genome Biology, 7, 212. 16677430 10.1186/gb-2006-7-4-212 PMC1557992

[pld381-bib-0042] Mirocha, C. J. , Gilchrist, D. G. , Shier, W. T. , Abbas, H. K. , Wen, Y. , & Vesonder, R. F. (1992). AAL toxins, fumonisins (biology and chemistry) and host‐specificity concepts. Mycopathologia, 117, 47–56. 1513374 10.1007/BF00497278

[pld381-bib-0043] Muro, I. , Hay, B. A. , & Clem, R. J. (2002). The Drosophila DIAP1 protein is required to prevent accumulation of a continuously generated, processed form of the apical caspase DRONC. Journal of Biological Chemistry, 277, 49644–49650. 12397080 10.1074/jbc.M203464200

[pld381-bib-0044] Nagashima, Y. , Mishiba, K. , Suzuki, E. , Shimada, Y. , Iwata, Y. , & Koizumi, N. (2011). Arabidopsis IRE1 catalyses unconventional splicing of bZIP60 mRNA to produce the active transcription factor. Scientific Reports, 1, 29. 22355548 10.1038/srep00029 PMC3216516

[pld381-bib-0045] Nelson, B. K. , Cai, X. , & Nebenfuhr, A. (2007). A multicolored set of in vivo organelle markers for co‐localization studies in Arabidopsis and other plants. The Plant Journal, 51, 1126–1136. 17666025 10.1111/j.1365-313X.2007.03212.x

[pld381-bib-0046] Noutoshi, Y. , Ito, T. , Seki, M. , Nakashita, H. , Yoshida, S. , Marco, Y. , … Shinozaki, K. (2005). A single amino acid insertion in the WRKY domain of the Arabidopsis TIR‐NBS‐LRR‐WRKY‐type disease resistance protein SLH1 (sensitive to low humidity 1) causes activation of defense responses and hypersensitive cell death. The Plant Journal, 43, 873–888. 16146526 10.1111/j.1365-313X.2005.02500.x

[pld381-bib-0047] Padmanabhan, M. S. , Ma, S. , Burch‐Smith, T. M. , Czymmek, K. , Huijser, P. , & Dinesh‐Kumar, S. P. (2013). Novel positive regulatory role for the SPL6 transcription factor in the N TIR‐NB‐LRR receptor‐mediated plant innate immunity. PLoS Pathogens, 9, e1003235. 23516366 10.1371/journal.ppat.1003235 PMC3597514

[pld381-bib-0048] Parrish, A. B. , Freel, C. D. , & Kornbluth, S. (2013). Cellular mechanisms controlling caspase activation and function. Cold Spring Harbor Perspectives in Biology, 5, 1–24. 10.1101/cshperspect.a008672 PMC3660825 23732469

[pld381-bib-0049] Preston, J. C. , & Hileman, L. C. (2013). Functional evolution in the plant SQUAMOSA‐promoter binding protein‐like (SPL) gene family. Frontiers in Plant Science, 4, 80. 23577017 10.3389/fpls.2013.00080 PMC3617394

[pld381-bib-0050] Qi, M. , Link, T. I. , Muller, M. , Hirschburger, D. , Pudake, R. N. , Pedley, K. F. , … Whitham, S. A. (2016). A small cysteine‐rich protein from the Asian soybean rust fungus, phakopsora pachyrhizi. Suppresses plant immunity. PLoS Pathogens, 12, e1005827. 27676173 10.1371/journal.ppat.1005827 PMC5038961

[pld381-bib-0051] Ranjan, A. , Jayaraman, D. , Grau, C. , Hill, J. H. , Whitham, S. A. , Ane, J. M. , … Kabbage, M. (2017). The pathogenic development of Sclerotinia sclerotiorum in soybean requires specific host NADPH oxidases. Molecular Plant Pathology, 19, 700–714. 28378935 10.1111/mpp.12555 PMC6638103

[pld381-bib-0052] RStudio Team (2016). RStudio: Integrated development for R. Boston, MA: RStudio, Inc.

[pld381-bib-0053] Ruifrok, A. C. , & Johnston, D. A. (2001). Quantification of histochemical staining by color deconvolution. Analytical and Quantitative Cytology and Histology, 23, 291–299. 11531144

[pld381-bib-0054] Sakamoto, M. , Tada, Y. , Nakayashiki, H. , Tosa, Y. , & Mayama, S. (2005). Two phases of intracellular reactive oxygen species production during victorin‐induced cell death in oats. Journal of General Plant Pathology, 71, 387–394.

[pld381-bib-0055] Salinas, M. , Xing, S. , Hohmann, S. , Berndtgen, R. , & Huijser, P. (2012). Genomic organization, phylogenetic comparison and differential expression of the SBP‐box family of transcription factors in tomato. Planta, 235, 1171–1184. 22160465 10.1007/s00425-011-1565-y

[pld381-bib-0056] Schindelin, J. , Arganda‐Carreras, I. , Frise, E. , Kaynig, V. , Longair, M. , Pietzsch, T. , … Cardona, A. (2012). Fiji: An open‐source platform for biological‐image analysis. Nature Methods, 9, 676–682. 22743772 10.1038/nmeth.2019 PMC3855844

[pld381-bib-0057] Shi, L. , Bielawski, J. , Mu, J. , Dong, H. , Teng, C. , Zhang, J. , … Zuo, J. (2007). Involvement of sphingoid bases in mediating reactive oxygen intermediate production and programmed cell death in Arabidopsis. Cell Research, 17, 1030–1040. 18059378 10.1038/cr.2007.100

[pld381-bib-0058] Slabaugh, E. , & Brandizzi, F. (2011). Membrane‐tethered transcription factors provide a connection between stress response and developmental pathways. Plant Signaling & Behavior, 6, 1210–1211. 21758012 10.4161/psb.6.8.16047 PMC3260725

[pld381-bib-0059] Stone, J. M. , Liang, X. , Nekl, E. R. , & Stiers, J. J. (2005). Arabidopsis AtSPL14, a plant‐specific SBP‐domain transcription factor, participates in plant development and sensitivity to fumonisin B1. The Plant Journal, 41, 744–754. 15703061 10.1111/j.1365-313X.2005.02334.x

[pld381-bib-0060] Trapnell, C. , Roberts, A. , Goff, L. , Pertea, G. , Kim, D. , Kelley, D. R. , … Pachter, L. (2012). Differential gene and transcript expression analysis of RNA‐seq experiments with TopHat and Cufflinks. Nature Protocols, 7, 562–578. 22383036 10.1038/nprot.2012.016 PMC3334321

[pld381-bib-0061] Verhagen, A. M. , Coulson, E. J. , & Vaux, D. L. (2001). Inhibitor of apoptosis proteins and their relatives: IAPs and other BIRPs. Genome Biology, 2, REVIEWS3009. 11516343 10.1186/gb-2001-2-7-reviews3009 PMC139420

[pld381-bib-0062] Wang, H. , & Wang, H. (2015). The miR156/SPL module, a regulatory hub and versatile toolbox, gears up crops for enhanced agronomic traits. Molecular Plant, 8, 677–688. 25617719 10.1016/j.molp.2015.01.008

[pld381-bib-0063] Williams, B. , Kabbage, M. , Kim, H.‐J. , Britt, R. , & Dickman, M. B. (2011). Tipping the balance: Sclerotinia sclerotiorum secreted oxalic acid suppresses host defenses by manipulating the host redox environment. PLOS Pathogens, 7, e1002107. 21738471 10.1371/journal.ppat.1002107 PMC3128121

[pld381-bib-0064] Wu, F.‐H. , Shen, S.‐C. , Lee, L.‐Y. , Lee, S.‐H. , Chan, M.‐T. , & Lin, C.‐S. (2009). Tape‐Arabidopsis Sandwich – a simpler Arabidopsis protoplast isolation method. Plant Methods, 5, 16. 19930690 10.1186/1746-4811-5-16 PMC2794253

[pld381-bib-0065] Yamasaki, K. , Kigawa, T. , Seki, M. , Shinozaki, K. , & Yokoyama, S. (2013). DNA‐binding domains of plant‐specific transcription factors: Structure, function, and evolution. Trends in Plant Science, 18, 267–276. 23040085 10.1016/j.tplants.2012.09.001

[pld381-bib-0066] Yang, Z. T. , Wang, M. J. , Sun, L. , Lu, S. J. , Bi, D. L. , Sun, L. , … Liu, J. X. (2014). The membrane‐associated transcription factor NAC089 controls ER‐stress‐induced programmed cell death in plants. PLoS Genetics, 10, e1004243. 24675811 10.1371/journal.pgen.1004243 PMC3967986

[pld381-bib-0067] Yoo, S. D. , Cho, Y. H. , & Sheen, J. (2007). Arabidopsis mesophyll protoplasts: A versatile cell system for transient gene expression analysis. Nature Protocols, 2, 1565–1572. 17585298 10.1038/nprot.2007.199

