# Supplementary material for: An inhibitor of apoptosis (SfIAP) interacts with SQUAMOSA promoter‐binding protein (SBP) transcription factors that exhibit pro‐cell death characteristics
Source: Plant Direct. 2018 Aug 10;2(8):e00081. doi: 10.1002/pld3.81 (PMC6508781; doi:10.1002/pld3.81)
Supplement: Supplementary file 1 [file PLD3-2-e00081-s001.pdf]

Supplementary Table S1: Summary of primers used in this study.

Target gene	Sequence	Purpose
SfIAP	fwd: 5'-CGGAATTCATGTGGTCGTGTTCCTTACCT-3'<br>rev: 5'-CGGGATCCTCACGAGAAATATAACCGCA-3'	Yeast two-hybrid
$SfIAP_{BIR1}$	fwd: 5'- CCGGAATTCATGAGTGCGGACACAGT-3'<br>rev: 5'- CCGCTCGAGTCATGCTGCAGTACCGTTC-3'	Yeast two-hybrid
pB42AD	fwd: 5'-TCATGAAATTGAAGCGGATGTT-3'<br>rev: 5'-CAACAACGTATCTACCAACGATTT-3'	Yeast two-hybrid
$SfIAP^{M4}$(I332A)	fwd: 5'-ATGAGAAAAGAGGATGAACGTATGAAAAC-3'<br>rev: 5'-TCACGAGAAATATAACCGCACTGC-3'	CoIP
SlySBP-like	fwd: 5'-ATGGCAAACAATGATGCTGG-3'<br>rev: 5'-TCATCCAGAGATGTCAGCAG-3'	Overexpression in *N. benthamiana*
SlySBP4	fwd: 5'-ATGGAAACAGCTAATAATCAGTT-3'<br>rev: 5'-TCAACGTAAGTGAAAGTTTTTGTG-3'	Overexpression in *N. benthamiana*
SlySBP6a	fwd: 5'-ATGGAGTCATGGAGCTATTTTTCAG-3'<br>rev: 5'-CTAAGTATGTCTTGGCATGCAAAG-3'	Overexpression in *N. benthamiana*
SlySBP6c	fwd: 5'-ATGGAGTCTTGGAGCTATGTC-3'<br>rev: 5'-TCATTTCATGGAACGTCTTTGATG-3'	Overexpression in *N. benthamiana*
SlySBP8b	fwd: 5'-ATGTTGGACTATGAATGGGAAAATC-3'<br>rev: 5'-TTAGTCACCCATTGGAAAAGAAG-3'	Overexpression in *N. benthamiana* / CoIP/localization
SlySBP12a	fwd: 5'-ATGGAAGCGAGTGTGG-3'<br>rev: 5'-TCAGCTTGTTCCAAAGTCC-3'	Overexpression in *N. benthamiana*/ CoIP/localization
SlySBP12a(ΔTMD)	fwd: SlySBP12a fwd primer<br>rev: 5'-TCATGATATCTCTAAGCTGGTGGTTG -3'	Overexpression in *N. benthamiana*/ localization
$TMD_{SlySBP12a}$	fwd: 5'-ATGACTGAGAGAAAAGCCATTCC-3'<br>rev: SlySBP12a rev primer	Overexpression in *N. benthamiana*/ localization
attB1 adaptor	5'-GGGGACAAGTTTGTACAAAAAAGCAGGCTGG-3'	Gateway^{TM} cloning
attB2 adaptor	5'-GGGGACCACTTTGTACAAGAAAGCTGGGTG-3'	Gateway^{TM} cloning

Supplementary Table S2: Genes involved in stress responses with an SBP cis-element in their promoter. LMM – lesion mimic mutant.

**Locus ID**	**Description**	**LMM**
At5g40440	TIR-NBS-LRR	no
At5g48770	TIR-NBS-LRR	no
At4g16860	RPP4	yes
At5g45260	RRS1 (WRKY 52)	yes
At2g35930	AtPUB23	no
At1g13960	WRKY 4	no
At4g01250	WRKY 22	no
At5g15130	WRKY 72	no
At1g79110	BOI-related gene 2 (BRG2)	no
At1g76580	Contains SBP-box domain	no

(A)

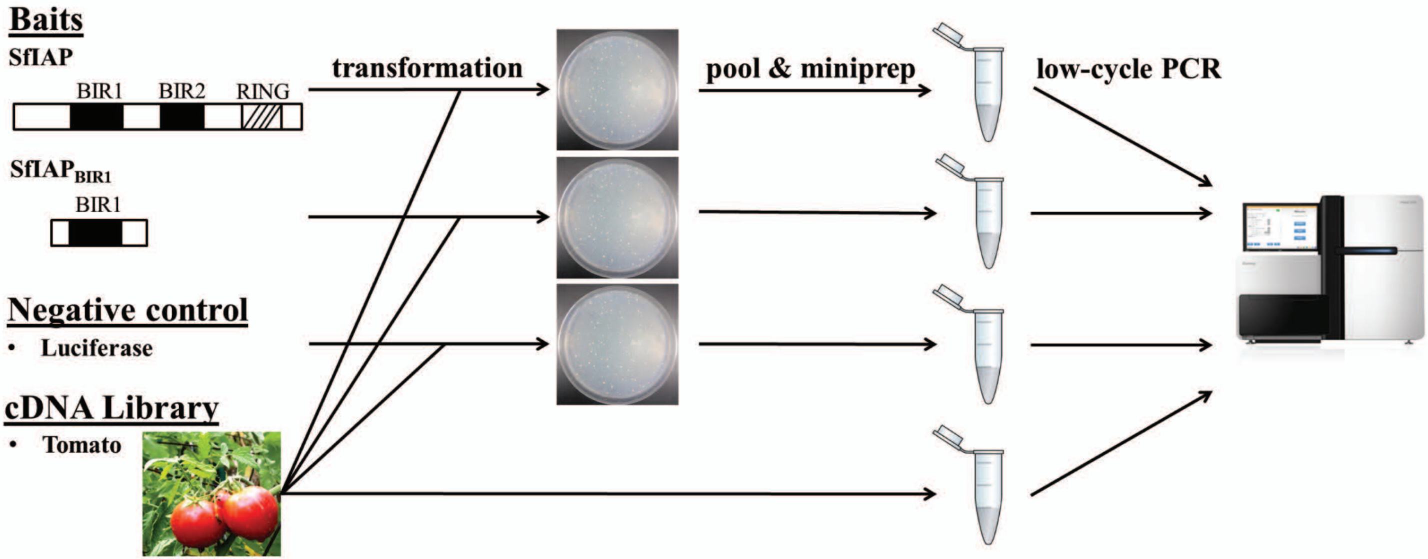

(B)

$$Enrichment = \frac{FPKM_{bait} - FPKM_{luciferase}}{FPKM_{bait} + FPKM_{luciferase} + FPKM_{library}} \times 100$$

Supplementary Fig. S1. Summary of the (A) QIS-Seq approach used to identify SfIAP-interacting partners and (B) the equation used to calculate enrichment scores for each gene.

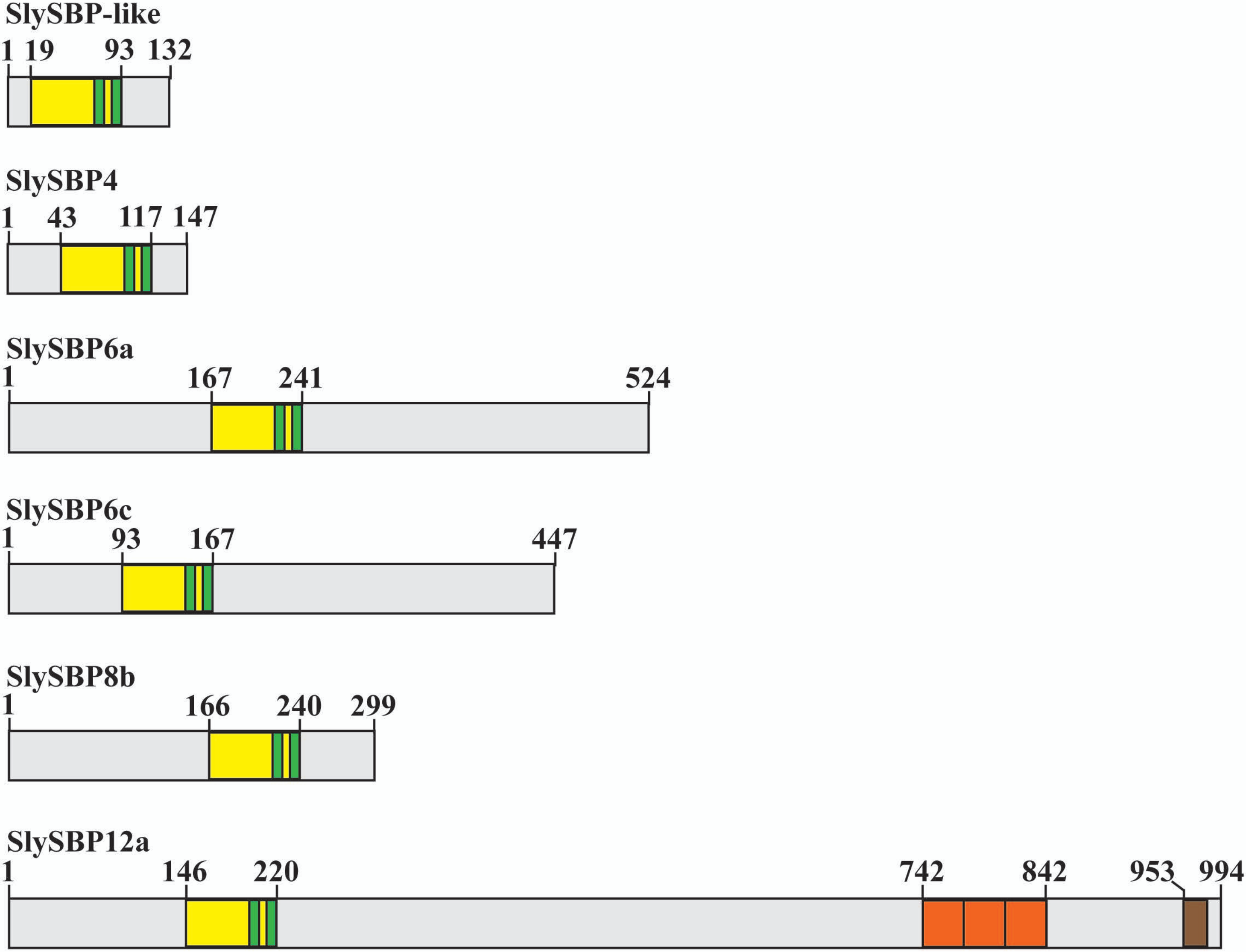

Supplementary Fig. S2. Diagram of SlySBP proteins overexpressed in *N. benthamiana*. Numbers represent amino acid positions. Yellow – SBP domain. Green – NLS. Orange – Ankyrin repeats. Brown – TMD. HMMER (hmmer.org) was used in conjunction with a Pfam database to identify conserved protein domains.

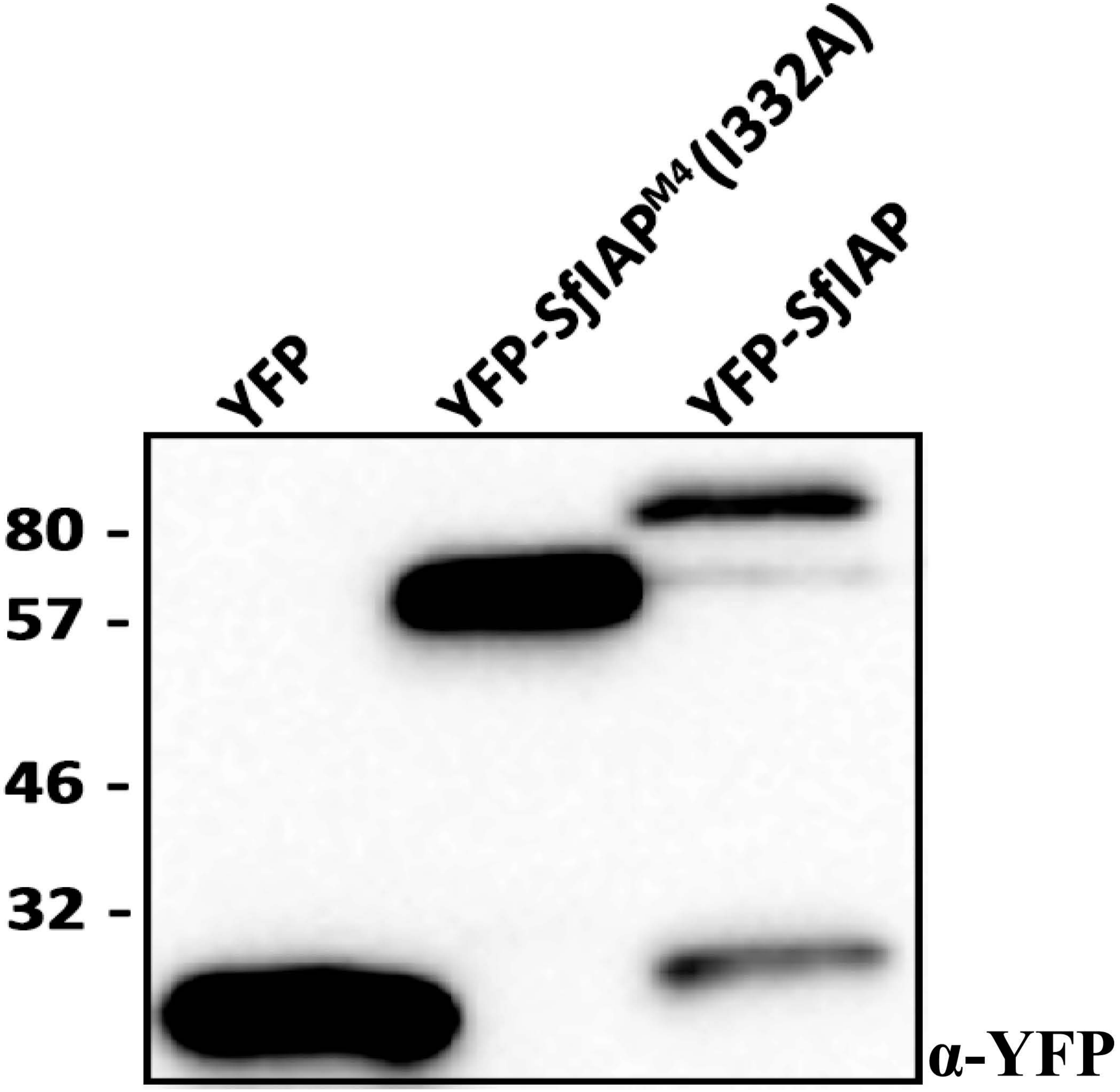

Supplementary Fig. S3. Western blot confirms SfIAP^{M4}(I332A) is not cleaved at its N-terminus. Leaves from *N. benthamiana* transiently transformed with *35S:YFP, 35S:YFP-SfIAP^{M4}(I332A),* or *35S:YFP-SfIAP* were collected 2 days post-transformation. An α-YFP antibody was used to detect protein accumulation.

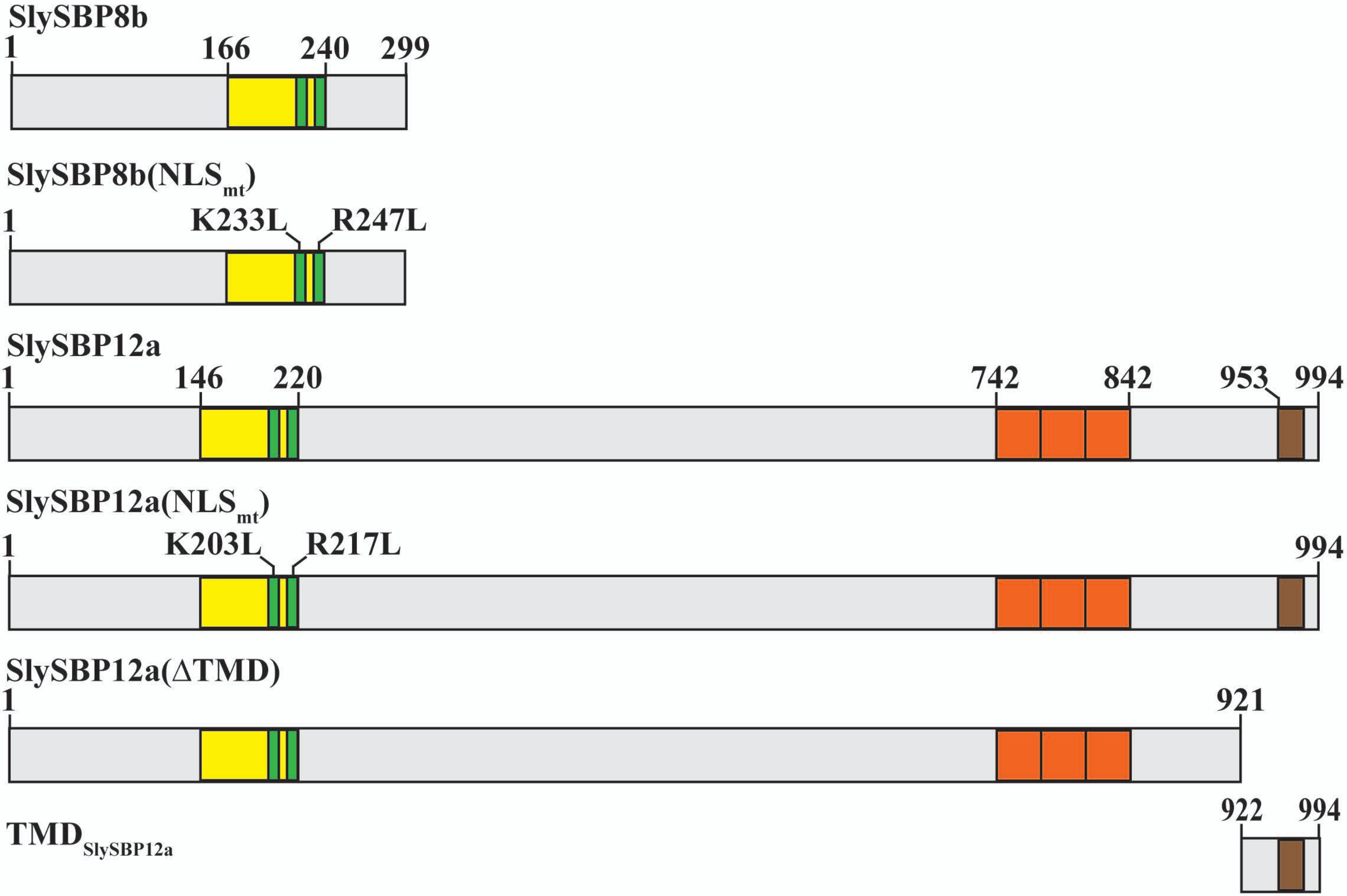

Supplementary Fig. S4. Diagram of wild-type and mutant SlySBP8b and -12a constructs used in this study. Numbers represent amino acid positions. Yellow – SBP domain. Green – NLS. Orange – Ankyrin repeats. Brown – TMD. HMMER (hmmer.org) was used in conjunction with a Pfam database to identify conserved protein domains.

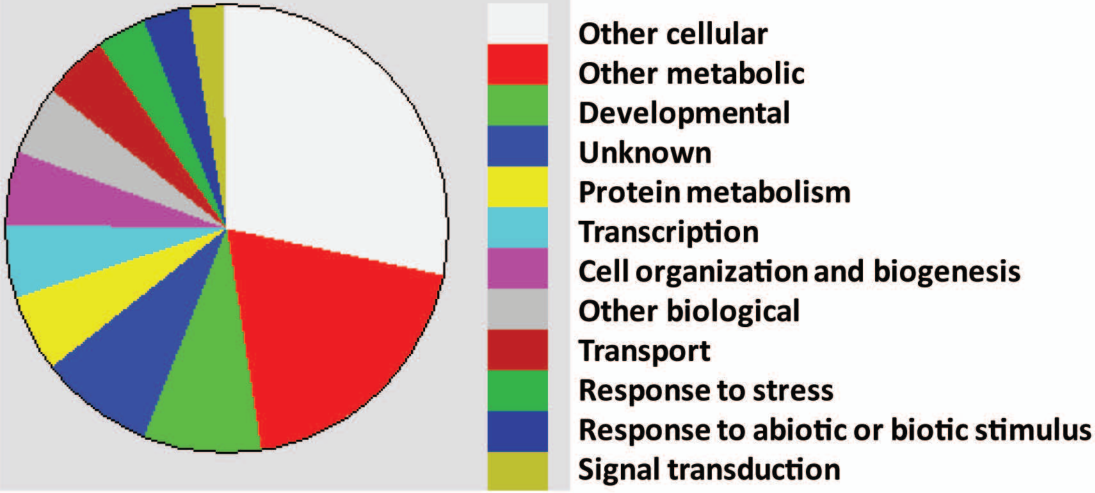

Supplementary Fig. S5. Pie chart summarizing the biological functions of genes containing the SBP cis-element in their promoter region.

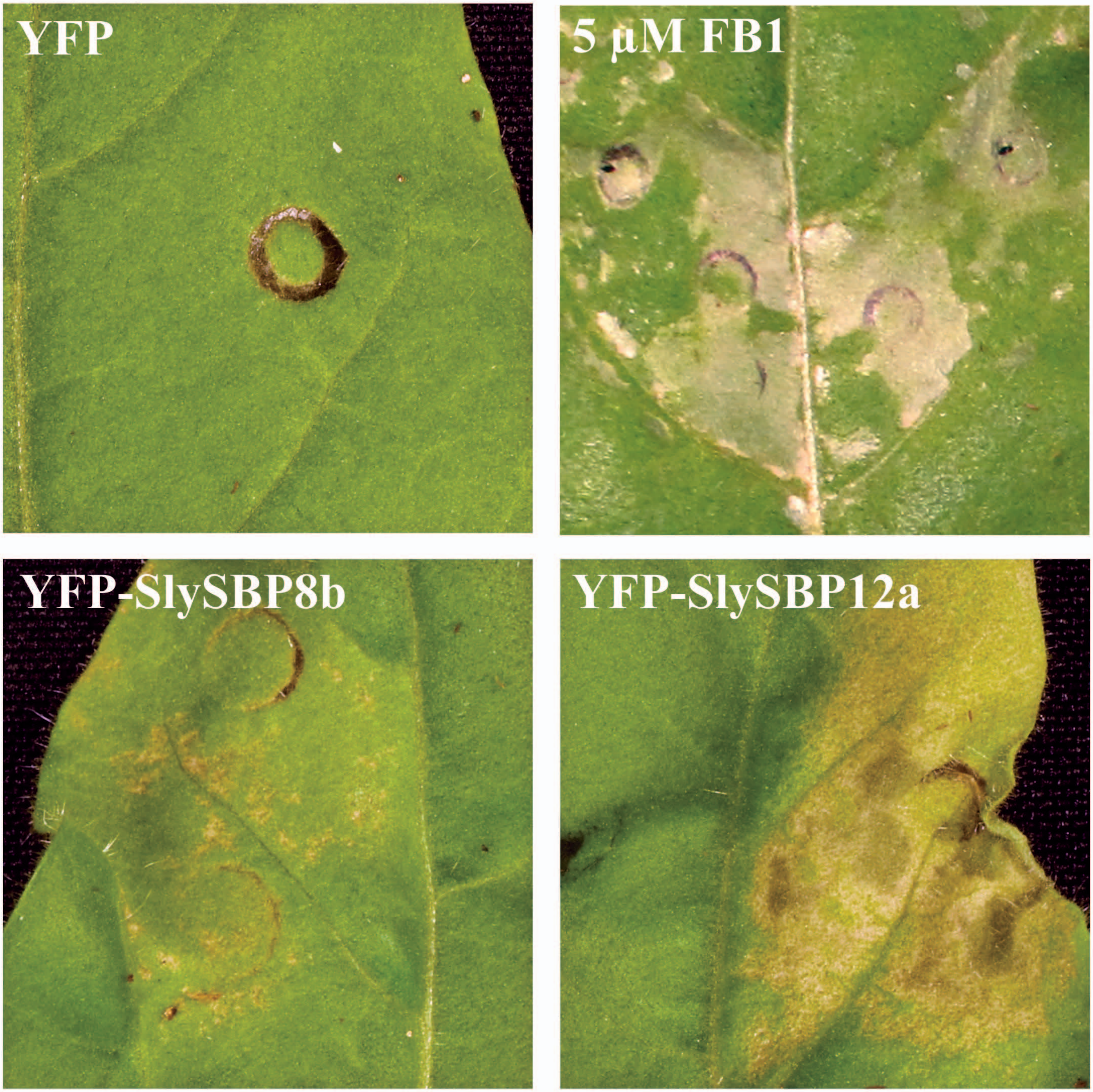

Supplementary Fig. S6. Cell death induced by overexpression of *SlySBP8b* and *SlySBP12a* in *N. glutinosa*. Leaves were transformed with *35S:YFP, 35S:YFP-SlySBP8b,* and *35S:YFP-SlySBP12a*. Leaves were treated with 5 μM FB1 by coinfiltrating with Agrobacterium carrying *35S:YFP.* Images of leaves were taken 5 days post-transformation.
