# Supplementary material for: An inhibitor of apoptosis (SfIAP) interacts with SQUAMOSA promoter‐binding protein (SBP) transcription factors that exhibit pro‐cell death characteristics
Source: Plant Direct. 2018 Aug 10;2(8):e00081. doi: 10.1002/pld3.81 (PMC6508781; doi:10.1002/pld3.81)
Supplement: Supplementary file 2 [file PLD3-2-e00081-s002.pdf]

Note: Line numbers correspond to the revised manuscript with track changes marked

Reviewer #1 :

This manuscript assigns a novel function in the initiation of programmed cell death to a class of transcription factors (SBP family) not previously known to have this role. A creative and original approach was used, namely screening for plant proteins that interact with an ectopically expressed animal protein (SfIAP) that protects against biotic and abiotic stress. The experiments are convincingly performed and the controls are adequate to make these points. Because this is an entirely novel approach with proven success in identifying previously unknown programmed cell death actors in plants, the work is of broad interest to the field and will likely stimulate much emulation in identifying further actors on these and related signaling pathways.
The major difficulty with this study is that it is unclear how these SBP factors are acting in the plant to mediate stress response, as the insect protein target (or any known homologue?) is apparently absent. Although the authors do makes some guesses based on their data (principally from sub cellular localisation) on how these SBP factors may function in stress response, it is not clear how they may interact with the existing cellular machinery. It would be helpful if they could provide a fuller description of the known actors involved in plant programmed cell death and discuss their data in this context. For instance, do any known actors have binding sites for the SBP factors in their promoter regions? Or do they themselves interact with sites on the promoters of SBP factors? Simple computer software programs can perform these kinds of analyses quite readily for Arabidopsis (i.e. identify whether there are binding sites for SBP factors in promoters of genes known to be implicated in plant programmed cell death and vice versa). Some ties to a known plant signaling mechanism involved in programmed cell death, or at least a more extended discussion of possible links, would strengthen the paper.

We thank the reviewer for this excellent suggestion. We have conducted a promoter analysis of Arabidopsis genes containing the canonical SBP binding site. The results of this analysis were included to the results section (Lines 352-360) and Supplemental Table 2 and Supplemental Figure 5 were added. In short, there are many genes that contain SBP binding sequences in their promoter and we were able to single out a few linked to cell death and stress response phenotypes (Supplemental Table 2). However, due to the large number of genes with predicted SBP-binding sites in their promoters, future studies will need to utilize additional tools such as ChIP-Seq to determine genes regulated by SlySBP8b and SlySBP12a *in vivo*. This was discussed in lines 521-537. Lines 246-251 were added to the Methods explaining the promoter analysis.

Reviewer #2

Title: An inhibitor of apoptosis (SfIAP) interacts with SQUAMOSA promoter binding protein (SBP) transcription factors that exhibit pro-cell death characteristics

Ryan Kessens[1], Nick Sorensen[1], Mehdi Kabbage[1*]

Comments to the author: The authors of this paper identified and demonstrated the role of two SQUAMOSA promoter binding protein (SBP) transcription factors (SlySBP8b and SlysSBP12a) in plant cell death regulation and resistance to necrotrophic pathogens. The

experiments and results in this paper support the conclusions, however, some experiments are missing key controls that need to be addressed before its publication.

Revisions require before publication

1- Fig 1: The following information need to be added in the methods: how many leaves, how many plants and how many spots were done. For example 3 plants/2 leaves/ 2 spots = 12 spots, thus 12/12 develop cell death for the genes and 0/12 for the YFP empty vector control. Also it will be helpful for the reviewers add a scheme of the Protein domains and structure of all the SBP TFs from this study as done in Sup. Fig.3 considering the different protein sizes in Fig. 1B. Are all those Full length proteins?, this information is not included in the Ms.
   a. A total of 10 leaves from 5 plants were scored for cell death for each of the SBP constructs tested. This has now been clarified in the Figure 1 legend and text was added to lines 289-298 of the Results. The number of leaves displaying a cell death phenotype were also added to Figure 1.
   b. All proteins expressed in Figure 1 were full-length as mentioned in the Materials and Methods section. However, we have now clarified this in line 283 of the Results. This was also added to the Figure 1 legend.
   c. As suggested by the reviewer, Supplementary Figure 2 was created to show readers the structure of all SBPs identified in the yeast two-hybrid and expressed in Figure 1. Text was added to lines 286-288 of the Results and a new figure legend was added.

2- Fig. 2. YFP-SfIAP full length should have been included as C-terminal fusion since the N-terminal is cleaved *in planta*. I wonder why the authors did not use C-terminal fusions rather than deleted N-terminal region M4 and mutate the Ring domain?
   a. The Y2H assay was performed before we were aware of processing of full-length SfIAP *in planta.* In order to best replicate the Y2H assay, in which genes contained N-terminal fusions of the Gal4 activation or DNA-binding domain, we decided it was best to utilize N-terminal fusions for the CoIP to avoid potential disruption of binding between bait and prey that may occur due to placement of a large tag such as YFP. If we had been aware of N-terminal processing before the Y2H we would have included a C-terminal fusion in our Y2H. Additionally, SfIAP^{M4}(I332A) contains all of the same domains (BIR1, BIR2, and RING) as SfIAP. The only difference is the I332A mutation abolishes E3 ligase activity. The purpose of mutating the RING domain was to abolish E3 ligase activity and prolong potential transient interactions. This is mentioned in line 305 "To account for the possibility that SfIAP may interact transiently with SlySBP12a, an E3 ligase mutant of the truncated SfIAP protein was generated by mutating a conserved residue in the RING domain (Cerio *et al.*, 2010)".

3- Fig3. The YFP-empty vector control need to be included in the localization studies in tomato protoplast as was done when expressed in *N. benthamiana*.

   a. The YFP-empty vector control has been added to Fig3 and the Fig3 legend has been edited to reflect this addition.

4- Fig 3 and Fig 4, The authors concluded that the nuclear localization of the SBP8b and SBP12a TF is required for cell death phenotype because the NLSmt does not show cell death phenotype and the protein is expressed, however is critical that the authors show that these NLSmt construct are not longer localized in the nucleus. The localization of these two NLSmt constructs should be shown in tomato or in N benthamiana to confirm that there are not longer in the nucleus.

   a. We thank the reviewer for this insightful suggestion. This experiment has since been performed. Nuclear localization is still observed with the NLSmt constructs (Figure 5). However, cytoplasmic localization is observed with SlySBP8b(NLSmt) while this is not observed with SlySBP8b (Figure 5). This suggests that nuclear import is hindered in the SlySBP8b(NLSmt) but not completely abolished. However, Birkenbihl *et al.*, 2005 showed that truncation of the NLS impaired both DNA-binding and nuclear import thus establishing the critical importance of this motif for SBP function and cell death induction.
   b. In this revised version of the manuscript, text was modified (lines 336-352) to address our observation that nuclear localization still occurred. Instead of saying nuclear localization or DNA binding is required for cell death induction, we make the broader claim that a functional SBP domain is required for cell death induction. The critical importance of the NLS in nuclear import and DNA binding makes it clear that the NLSmt has a defective SBP domain. This was also addressed in Lines 509-519 of the Discussion.

5- Fig. 5 similar as figure 1: The following information needs to be added in the methods: how many leaves, how many plants and how many spots were done. For example 3 plants/2 leaves/ 2 spots = 12 spots, thus 12/12 develop cell death for the genes and 0/12 for the empty vector control which is also missing in this experiment.

   a. We have now quantified cell death caused by overexpression of the WT and NLSmt constructs (Figure 4B and 4C). Quantification of cell death was performed using electrolyte leakage and a free YFP negative control was included. For each biological replicate, 8 leaf discs were collected from two leaves on the same plant and pooled into a single well of a 12-well plate. A total of at least 13 biological replicates were used for each construct (line 202-203 and Figure 4 legend).

6-Fig: 8 DAB staining. In the previous experiments the SBP8b showed stronger cell death phenotype than SBP12a (Fig 1 and Fig 4) and in the DAB staining

experiments seems to be the opposite and there is similar accumulation of ROS when compared both genes. I suggest that the authors complement this with an electro-leakage measurement between two genes as done in Fig. 6B.

a. Electrolyte leakage assays have now been performed. See Figure 4B and 4C.

7-Fig. 9. Considering the marginal effects on lesion size after pathogen inoculation, I will suggest conduct another quantitative measure to assess this phenotype as qPCR biomass quantification in these plants. In addition, the authors mentioned that the proteins are expressed but there is not a western blot or YFP- pictures from the fluorescence microscope as it was indicated in line 385 (Sup. Fig. 4). In sup. Fig. 4 again the SBP 8b cell death phenotype is weaker and SBP12a is stronger opposite to Fig 1 and fig. 4. Information of number of plants/leaves/spots is missing. Electro leakage experiments need to be conducted at least in two different plants not only different leaves and using one plant

a. We recognize that these are marginal effects, but considering the large sample size and stringency of the blind and randomized method we are confident that these are true effects. For this reason, we do not believe qPCR will prove to be any more effective.
b. Cell death induction caused by overexpression of *YFP-SlySB8b* and *YFP-SlySBP12a* in *N. glutinosa* is evidence that these genes are being expressed as shown in Supplemental Fig. 6.
c. While we checked for gene expression using fluorescence microscopy, we did not deem it necessary to include this data as only leaves expressing the transgenes were included in the experiment. This would also require showing fluorescent images of all 54 replicates to prove we only used leaves expressing the transgenes. Additionally, the constructs we used were shown to be expressed in Figures 5 and 6.
d. The Figure 10 legend (previously Figure 9) specifies that “4 randomized and blind experiments were pooled representing 54 leaves for each treatment”. Line 421 also notes the number of experiments and sample size that was used. Lines 146-147 state that “*Nicotiana glutinosa* plants were infiltrated at 5-6 weeks of age with a single leaf being used on each plant, typically corresponding to the 4th or 5th true leaf”. This means the 54 biological replicates represent 54 different plants with a single leaf being used from each plant.
